# The estrogen receptor variants β2 and β5 induce stem cell characteristics and chemotherapy resistance in prostate cancer through activation of hypoxic signaling

**DOI:** 10.18632/oncotarget.26345

**Published:** 2018-11-20

**Authors:** Michelle Faria, Peter Shepherd, Yinghong Pan, Sujash S. Chatterjee, Nora Navone, Jan-Åke Gustafsson, Anders Strom

**Affiliations:** ^1^ University of Houston, Department of Biology and Biochemistry, Center for Nuclear, Receptors and Cell Signaling, Science and Engineering Research Center, Houston, Texas, USA; ^2^ Department of Genitourinary Medical Oncology, The University of Texas MD Anderson Cancer Center, Houston, Texas, USA; ^3^ Department of Biosciences and Nutrition, Karolinska Institutet, Novum, Huddinge, Sweden

**Keywords:** HIF, chemotherapy resistance, HOTAIR, ERbeta2, ERbeta5

## Abstract

Chemotherapy resistant prostate cancer is a major clinical problem. When the prostate cancer has become androgen deprivation resistant, one of the few treatment regimens left is chemotherapy. There is a strong connection between a cancer’s stem cell like characteristics and drug resistance. By performing RNA-seq we observed several factors associated with stem cells being strongly up-regulated by the estrogen receptor β variants, β2 and β5. In addition, most of these factors were also up-regulated by hypoxia. One mechanism of chemotherapy resistance was expression of the hypoxia-regulated, drug transporter genes, where especially ABCG2 and MDR1 were shown to be expressed in recurrent prostate cancer and to cause chemotherapy resistance by efficiently transporting drugs like docetaxel out of the cells. Another mechanism was expression of the hypoxia-regulated Notch3 gene, which causes chemotherapy resistance in urothelial carcinoma, although the mechanism is unknown. It is well known that hypoxic signaling is involved in increasing chemotherapy resistance. Regulation of the hypoxic factors, HIF-1α and HIF-2α is very complex and extends far beyond hypoxia itself. We have recently shown that two of the estrogen receptor β variants, estrogen receptor β2 and β5, bind to and stabilize both HIF-1α and HIF-2α proteins leading to expression of HIF target genes. This study suggests that increased expression of the estrogen receptor β variants, β2 and β5, could be involved in development of a cancer’s stem cell characteristics and chemotherapy resistance, indicating that targeting these factors could prevent or reverse chemotherapy resistance and cancer stem cell expansion.

## INTRODUCTION

Prostate cancer is one of the leading causes of cancer deaths in men, despite the fact that many cases can be cured by early detection and surgery. One in four men diagnosed with prostate cancer eventually dies from the cancer. Most men respond to androgen deprivation therapy (ADT) or chemotherapy, but resistance usually occurs after 18–24 months [1]. A common treatment for castration resistant prostate cancer (CRPC) is the chemotherapy agent, docetaxel, which unfortunately leads to chemotherapy resistance over time. This condition has been linked to stem cell characteristics of the cancer [2] and these characteristics were shown to be induced by hypoxia [3]. The transporter proteins ABCG2 and MDR1 cause chemotherapy resistance in prostate cancer, where both genes were shown to be increased during hypoxia and are direct targets of HIF-1α [4, 5]. In 1996 a second estrogen receptor was discovered and named estrogen receptor β (ERβ) [6], two years later in 1998, a splice variant of ERβ, ERβ2, was cloned. This variant has a truncated ligand-binding domain (LBD) and a spliced-in unique exon [7]. The variant, which is primate- specific, cannot bind to estrogen or to a classical estrogen response element (ERE). Later, other variants were cloned like ERβ4 and ERβ5, all with a truncated LBD, spliced-in unique exon and unable to bind estrogen. No known function in normal physiology has been described for the variants, which have a low or non-existing expression in adult tissues indicating a function during development. These variants have been considered as non-functional and dependent on ERβ1 for their function. Recently we have shown that they bind to and stabilize HIF-1α and HIF-2α in prostate cancer cells, thus activating hypoxic signaling under normoxic conditions and in addition shown that they are recruited to HIF-1α response elements in chromatin [8]. The full length receptor, ERβ1 has on the other hand been shown to decrease HIF-1α signaling by up-regulating expression of prolyl dehydrogenase 2 (PDH2) in prostate [9]. Furthermore, ERβ1 has been shown to inhibit NF-kB signaling by causing down regulation of IKKβ, through decrease of HIF-1α level [10].

Previous clinical studies have shown that Selective Estrogen Receptor Modulators (SERM’s) like tamoxifen and raloxifene can improve the prognosis of aggressive prostate cancer, although the mechanism is unclear [11–14]. Our data indicated that 4OH tamoxifen reduced the increased proliferation caused by ERβ2, providing a possible explanation for the observed clinical effect. This study describes the effects of ERβ variants, ERβ2 and ERβ5, in prostate cancer showing them as possible drivers of chemotherapy resistance as well as cancer stem cell drivers.

## RESULTS

### The transcriptomes of ERβ2 and ERβ5 expressing PC3 cells showed a large overlap, but differences were also evident in both cases; there was activation of hypoxic signaling and induction of stem cell characteristics

**Figure 1:**
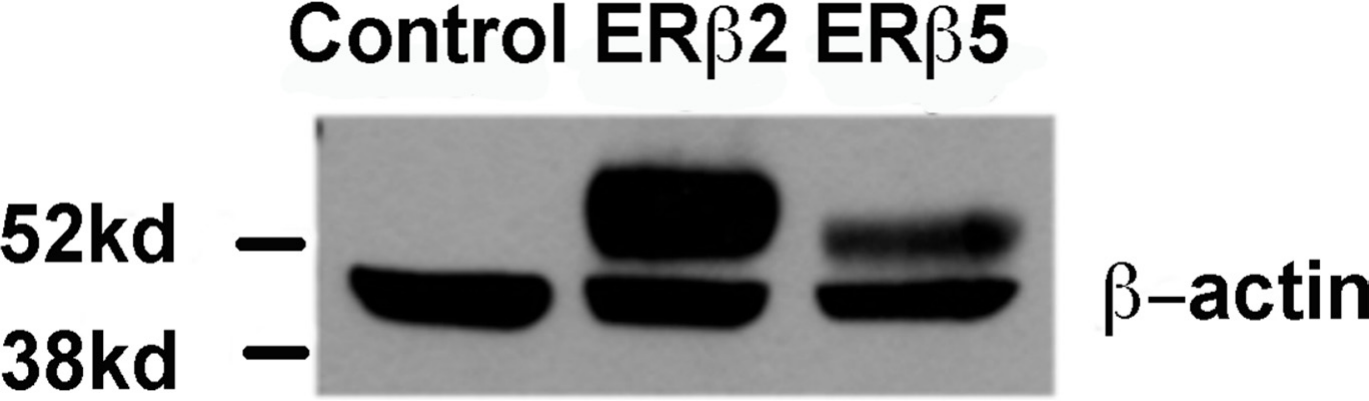
Western blot showing expression of ERβ2 and ERβ5 where the first lane is control transfected (empty transposon system without variant cDNA), then ERβ2 or ERβ5 stably transfected PC3 cells in lanes 2 and 3 30 µg of protein was separated on SDS-PAGE and the variants were detected using 14C8 N-terminal antibody which is detecting pan ERβ (all variants). All western blots are performed in the absence of doxycycline with expression of the variants at maximum level.

**Figure 2:**
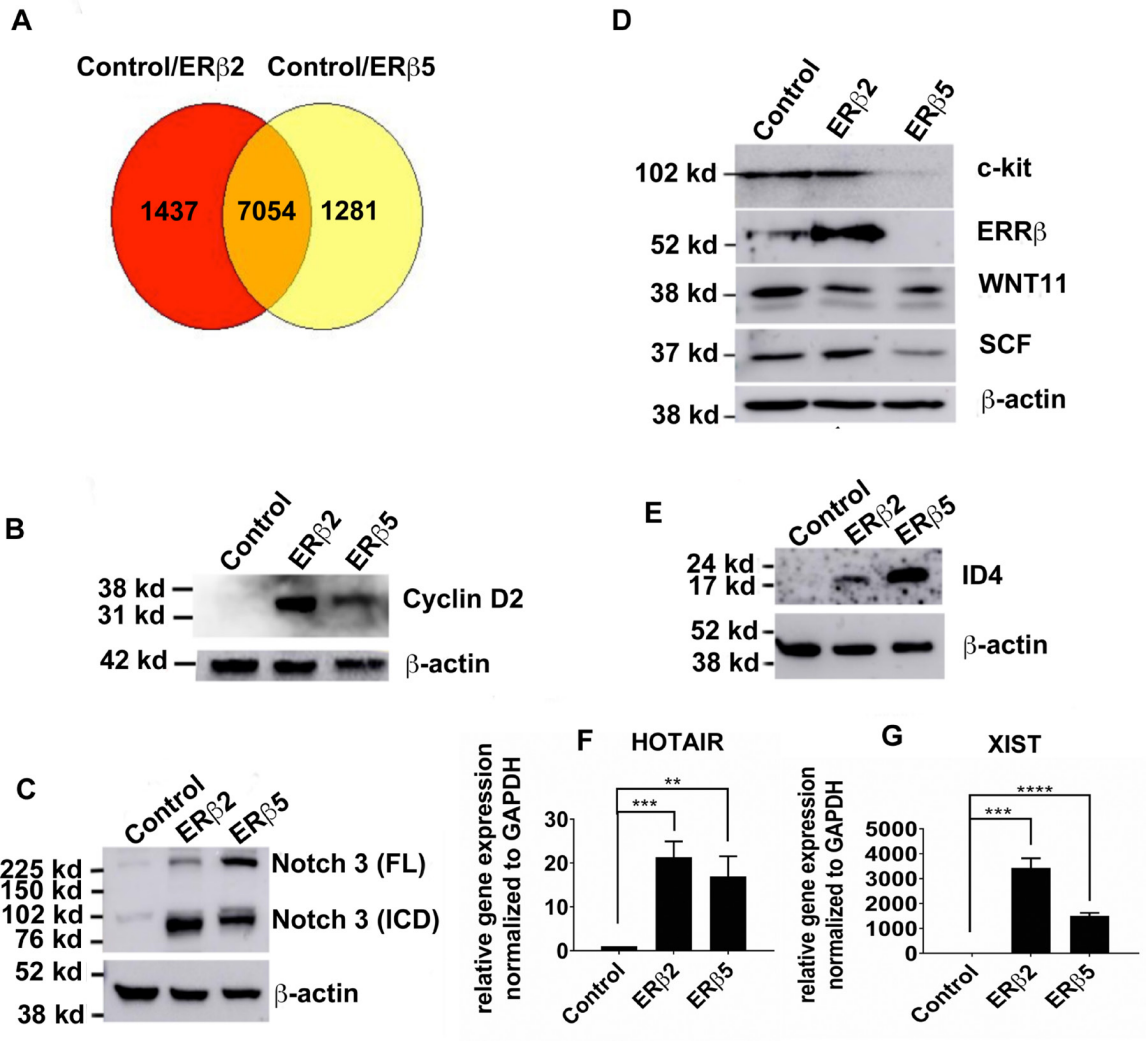
(**A**) Venn diagram showing ERβ2 and ERβ5 regulated transcripts and mutually regulated transcripts. Western blots of protein extracts from the stably expressing PC3 cells (**B**) Cyclin D2, (**C**) Notch3 Fullength (FL), Intracellular domain (ICD), (**D**) c-kit, ERRβ, WNT11, and SCF, (**E**) ID4. (**F**) qPCR of lncRNA HOTAIR (**G**) qPCR of lncRNA XIST.

To identify the full transcriptomes affected by ERβ2 and ERβ5 and to compare them with each other, we performed RNA-seq on PC3 cells stably expressing both variants compared to control PC3 cells (Figure 1). Two repeated samples showed high similarity in principal component analysis and in the heatmap (Supplementary Figures 1 and 2). There were 7054 mutually regulated transcripts, 1437 only regulated by ERβ2 and 1281 only regulated by ERβ5 (Figure 2A). Top diseases and functions for ERβ2 and ERβ5 included cellular movements, and cell cycle as highly represented themes (Table 1A and 1B). A preliminary analysis shows that among highly regulated mutual transcripts were factors involved in stem cell proliferation, like c-kit [15–17], ERRβ [18], WNT11 [19, 20], Notch3 [21], HOTAIR [22, 23], STAT3 [24], ID4 [25] and cyclin D2 [26, 27] (see Figure 2B–2E for western blots). In addition to the lncRNA HOTAIR, (Figure 2F) there was a strong regulation of the lncRNA XIST [28] (see Figure 2G). Many of these transcripts have previously been shown to be regulated by hypoxia, in agreement with the theory that hypoxia causes appearance of a stem cell phenotype [3]. We found that WNT11 protein was down regulated by the ERβ splice variants, although the transcript was highly induced, indicating a feedback loop, where the protein represses its mRNA levels i.e. a higher turnover of the protein could cause less repression of mRNA stability or transcription. Alternatively, a lower protein level within the cell indicated a higher rate of secretion out of the cell. Although c-kit was strongly regulated at the transcript level, we didn’t see any change at the protein level between control and ERβ2, and a reduction was observed with ERβ5, indicating that the protein was regulated by another pathway rather than through mRNA levels. The ligand for c-kit, SCF, was regulated in the same way, not changed between control and ERβ2, but reduced by ERβ5. For ERRβ an increased protein level was seen only for ERβ2, but a reduction was observed for ERβ5. As a result of increased Notch signaling, we saw high expression of HES5 and HEY2 in the RNAseq data. HES5 has been shown to increase activity of STAT3, which we also observed as being increased transcriptionally. STAT3 has furthermore been shown to play a central role in maintenance of a stem cell phenotype in glioblastoma [24]. Pathway analysis showed that both variants activated MAPK1 signaling (Supplementary Figure 3A and 3B). For a selection of highly regulated, mutual and variant- specific transcripts, see Supplementary Table 1A–1F. The complete lists with *p*-value and FDR is given in Supplementary Tables 4–8.

**Table 1A:** Top diseases and functions affected by ERβ2 from analysis of RNA-seq data

Score	Focus molecules	Top diseases and functions	
26	35	Connective Tissue Disorders, Inflammatory Disease, Inflammatory Response	
26	35	Cell Death and Survival, Auditory Disease, Auditory and Vestibular System Development and Function	
23	34	Cell Cycle, Organismal Injury and Abnormalities, Cellular Response to Therapeutics	
23	34	Cell Morphology, Cellular Development, Cellular Growth and Proliferation	
23	34	Organismal Injury and Abnormalities, Cancer, Dermatological Diseases and Conditions	
23	34	Cellular Movement, Cellular Assembly and Organization, Cell Morphology	
23	34	Tissue Development, Cellular Movement, Cardiovascular System Development and Function	
23	34	Cell Signaling, Antimicrobial Response, Inflammatory Response	
22	33	Skeletal and Muscular System Development and Function, Embryonic Development, Organismal Development	
22	33	Cancer, Organismal Injury and Abnormalities, Cellular Development	
22	33	Gene Expression, Cardiovascular System Development and Function, Organ Morphology	
22	33	Cardiac Enlargement, Cardiovascular Disease, Cardiovascular System Development and Function	
20	32	Cellular Movement, Immune Cell Trafficking, Gastrointestinal Disease	
20	32	Hematological System Development and Function, Humoral Immune Response, Lymphoid Tissue Structure and Development	
20	32	Embryonic Development, Nervous System Development and Function, Organ Development	
20	32	Cell Morphology, Cellular Movement, Organismal Injury and Abnormalities	
20	32	Cell Death and Survival, Organismal Injury and Abnormalities, Cellular Function and Maintenance	
20	32	Infectious Diseases, Respiratory Disease, Connective Tissue Development and Function	
20	32	Cellular Development, Hematological System Development and Function, Lymphoid Tissue Structure and Development	
18	31	Cell Death and Survival, Infectious Diseases, Tissue Morphology	
18	31	Cellular Movement, Cellular Function and Maintenance, Molecular Transport	
18	31	Cellular Movement, Cellular Development, Cellular Growth and Proliferation	
18	31	Cellular Movement, Cardiovascular System Development and Function, Immune Cell Trafficking	
18	31	Cell Death and Survival, Organismal Injury and Abnormalities, Cellular Development	
18	31	Auditory Disease, Neurological Disease, Hereditary Disorder	

**Table 1B:** Top diseases and functions affected by ERβ5 from analysis of RNA-seq data

Score	Focus molecules	Top diseases and functions	
26	35	Embryonic Development, Nervous System Development and Function, Organ Development	
26	35	Connective Tissue Disorders, Developmental Disorder, Endocrine System Disorders	
24	34	Organismal Injury and Abnormalities, Cell Cycle, Cellular Response to Therapeutics	
24	34	Cardiac Arteriopathy, Cardiovascular Disease, Organismal Injury and Abnormalities	
24	34	Tissue Development, Endocrine System Development and Function, Small Molecule Biochemistry	
24	34	Antimicrobial Response, Inflammatory Response, Cell Signaling	
22	33	Skeletal and Muscular System Development and Function, Embryonic Development, Organismal Development	
22	33	Cell Death and Survival, Cellular Development, Connective Tissue Development and Function	
22	33	Cancer, Organismal Injury and Abnormalities, Endocrine System Disorders	
22	33	Cell Cycle, Digestive System Development and Function, Organ Morphology	
22	33	Cardiovascular Disease, Organismal Injury and Abnormalities, Cardiovascular System Development and Function	
20	32	Cellular Movement, Cell Death and Survival, Cancer	
20	32	Infectious Diseases, Hematological Disease, Hematological System Development and Function	
20	32	Cellular Movement, Cellular Development, Connective Tissue Disorders	
20	32	Cellular Movement, Embryonic Development, Hair and Skin Development and Function	
20	32	Cell Death and Survival, Cellular Compromise, Cell Signaling	
20	32	Cellular Movement, Cellular Development, Cellular Growth and Proliferation	
20	32	Cellular Development, Skeletal and Muscular System Development and Function, Tissue Development	
20	32	Cardiovascular Disease, Cellular Assembly and Organization, Small Molecule Biochemistry	
20	32	Cellular Assembly and Organization, Hematological Disease, Immunological Disease	
20	32	Infectious Diseases, Inflammatory Disease, Organismal Injury and Abnormalities	
19	31	Cardiovascular System Development and Function, Organismal Development, Cellular Movement	
19	31	Connective Tissue Development and Function, Connective Tissue Disorders, Organismal Injury and Abnormalities	
19	31	Cellular Development, Cellular Growth and Proliferation, Connective Tissue Development and Function	
19	31	Cancer, Organismal Injury and Abnormalities, Gastrointestinal Disease	

### Expression of ERβ2 and ERβ5 induced resistance to chemotherapy in PC3 cells

**Figure 3:**
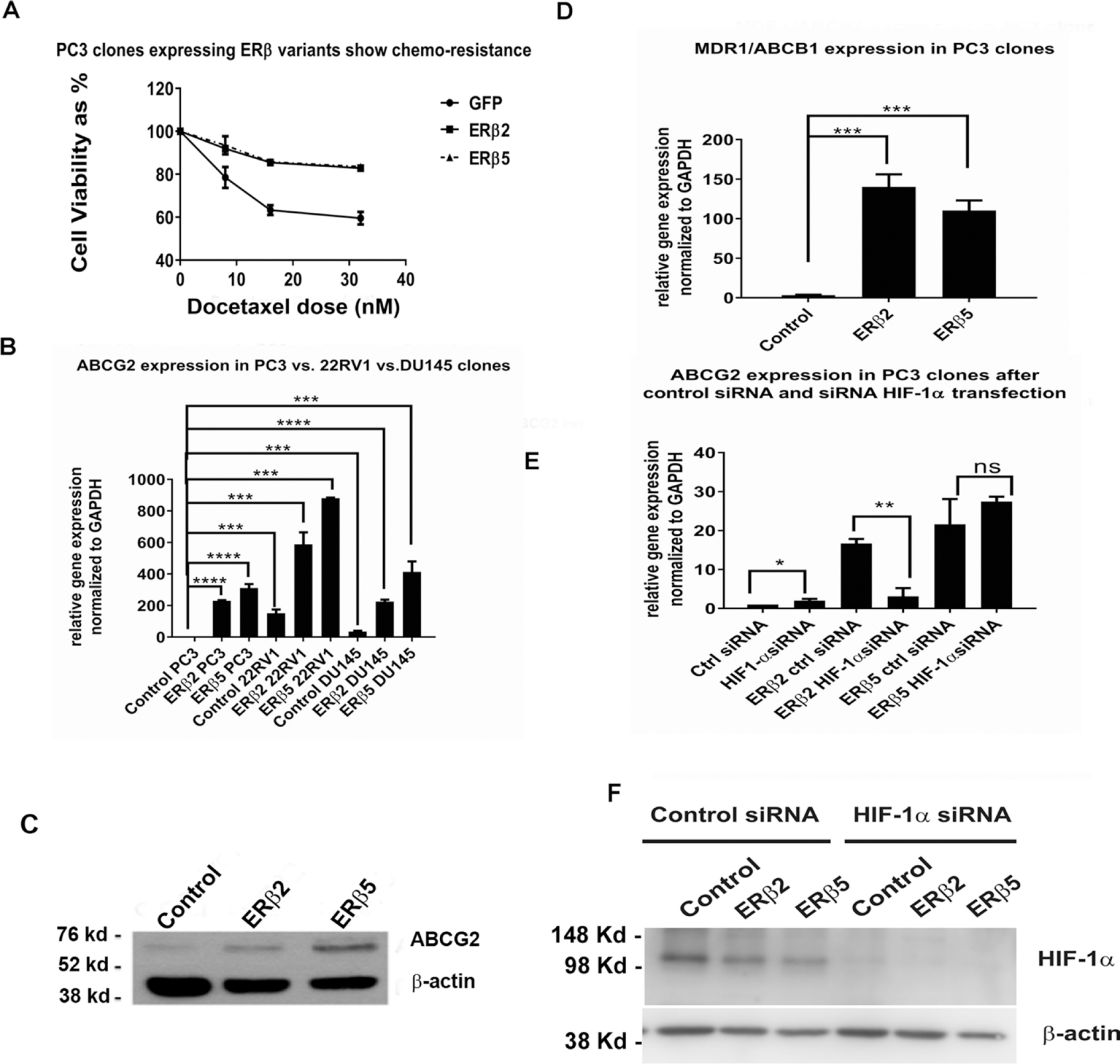
(**A**) PC3 cells expressing the variants show more chemotherapy resistance than control cells. 1.5 × 10^4^ cells were plated onto each well in a 96 well plate in 100 µl of media containing increasing doses of docetaxel. Cells were incubated at 37° C, 5% CO_2_ for 48 hours and cell viability was assayed using MTS assay. (**B**) qPCR of ABCG2 in three prostate cancer cell lines stably expressing the variants (for expression levels of the variants see Supplementary Figure 4). (**C**) Western blot of ABCG2 protein in PC3 cells stably expressing ERβ2 or ERβ5 compared to control PC3 cells. (**D**) qPCR of ABCB1/MDR1 in PC3 cells stably expressing ERβ2 or ERβ5 compared to control PC3 cells. (**E**) HIF-1α siRNA and siLUC siRNA are transfected into PC3 cells expressing ERβ2 or ERβ5, expression of ABCG2 is determined using qPCR. (**F**) Western blot of hypoxia induced HIF-1α protein after transfection of control siLUC and HIF-1α siRNA.

From the RNA-seq data, we observed that a number of genes involved in chemotherapy resistance and stem cell self-renewal were increased by expression of ERβ2 or ERβ5. We investigated whether PC3 cells expressing the ERβ splice variants were more chemotherapy resistant than control PC3 cells. We used docetaxel, which is a commonly used chemotherapeutic agent for advanced prostate cancer. We observed that cells expressing either of the variants were more resistant to treatment with docetaxel (Figure 3A). The Multi Drug Resistance (MDR) gene, ABCG2, previously shown to cause resistance to docetaxel in prostate [29] was regulated by ERβ2 and ERβ5 in three different prostate cancer cell lines (Figure 3B). In addition, we showed that ABCG2 was regulated at the protein level in PC3 cells (Figure 3C). Another MDR gene, MDR1/ABCB1, was more prominently regulated by the variants in PC3 cells (Figure 3D). We investigated whether HIF-1α was crucial for variant induction of ABCG2 by using siRNA to HIF-1α. This was accompanied with a markedly reduced induction of ABCG2 by ERβ2, while the induction by ERβ5 was unaffected; although the protein level of HIF-1α was dramatically reduced by the siRNA (Figure 3E). To validate functionality of the siRNA, we exposed the PC3 stable cells to hypoxia with and without transfection of HIF-1α siRNA, and found that the siRNA strongly reduced the HIF-1α protein level (Figure 3F).

### IKKβ activity was important for ERβ2 and ERβ5 induction of ABCG2

**Figure 4:**
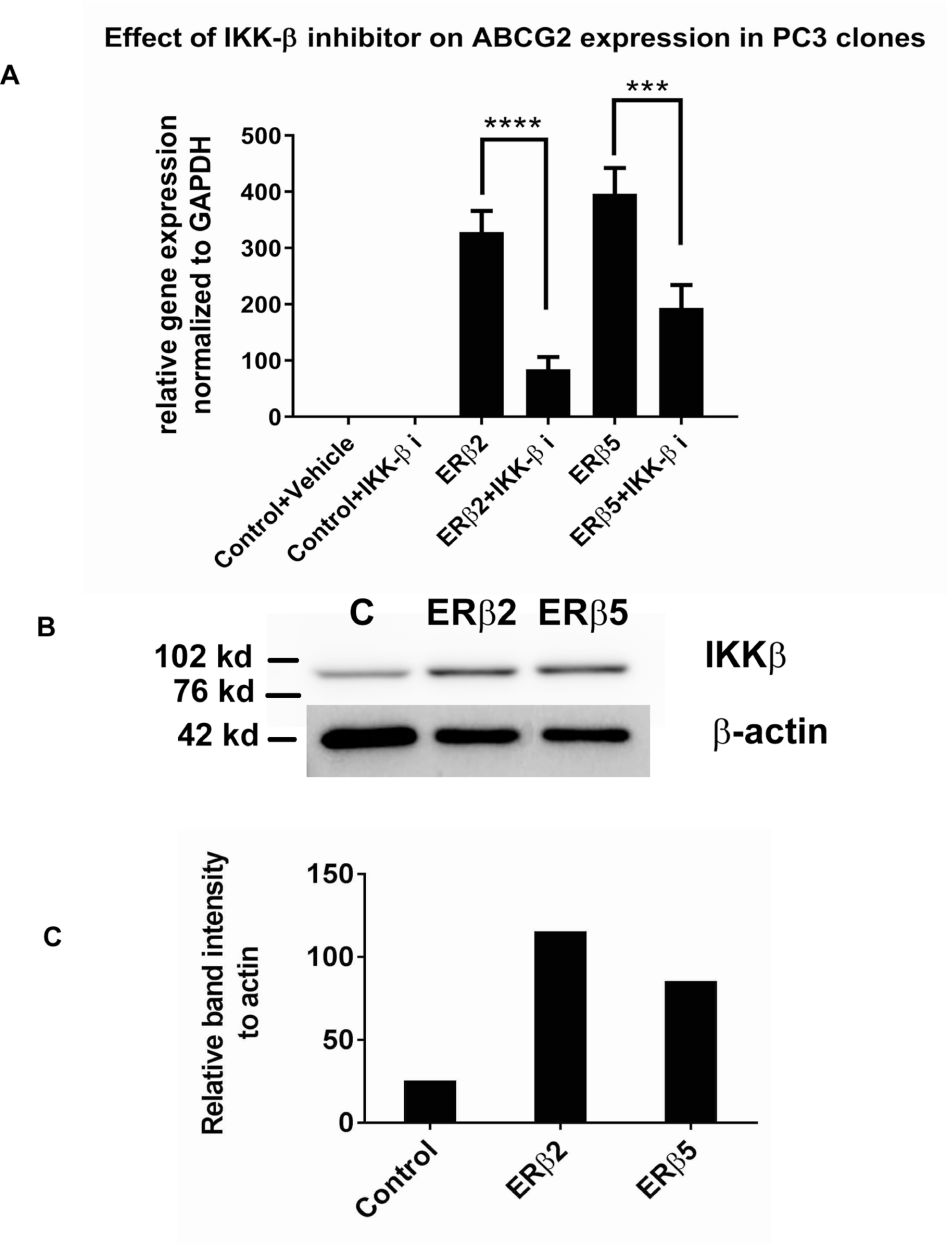
(**A**) Expression of ABCG2 is dependent on IKKβ activity. PC3 cells expressing ERβ2 or ERβ5 were exposed to 1 µM of the IKKβ inhibitor IMD-0354 for 24 hours and ABCG2 expression level was measured using real time PCR. (**B**) Western blot showing that IKKβ protein level is increased by expression of the variants. (**C**) Quantification of IKKbeta protein expression in PC3 stable cells versus GFP control.

ABCG2 has previously been shown to be regulated by NF-κB signaling. Hence, we used an inhibitor of the NF-κB activator, IKKβ, to investigate whether this factor is involved in ABCG2 induction by ERβ2 and ERβ5. We found that ABCG2 induction is reduced by addition of a specific IKKβ inhibitor to the ERβ2 and ERβ5-expressing PC3 cells (Figure 4A). In addition, we showed that IKKβ was upregulated at the protein level by ERβ2 and ERβ5 (Figure 4B and 4C).

### The ABCG2 inhibitor, YHO-13351, reduced ERβ2 and ERβ5 - induced resistance to chemotherapy

**Figure 5:**
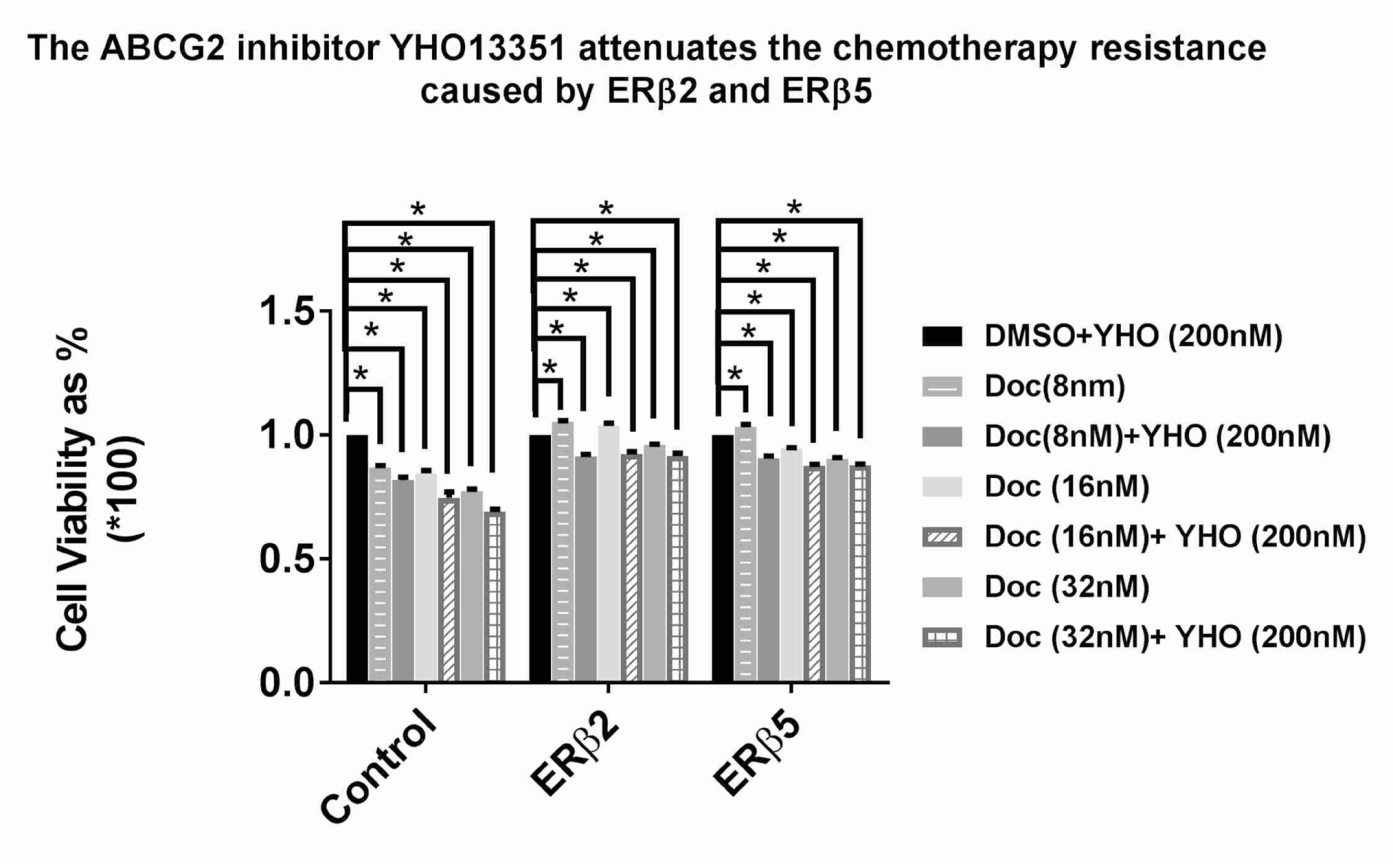
PC3 cells (control, ERβ2 and ERβ5) plated into a 96 well plate were subjected to chemotherapy treatment with docetaxel of increasing doses from 0, 8, 16, and 32 nM in the absence and presence of the ABCG2 inhibitor YHO-13351 (200 nM) MTS assay was performed after 48 hours.

ABCG2 has earlier been shown to cause chemotherapy resistance in prostate cancer [29]. We decided to use the ABCG2 inhibitor, YHO-13351, to investigate if ABCG2 mediated the observed chemotherapy resistance caused by ERβ2 and ERβ5. Co-treatment with docetaxel and YHO-13351 showed a slight reduction of chemotherapy resistance indicating that multiple pathways are likely involved in causing the chemotherapy resistance (Figure 5).

### ERβ2 and ERβ5 interacted with both HIF-1α and HIF-2α

**Figure 6:**
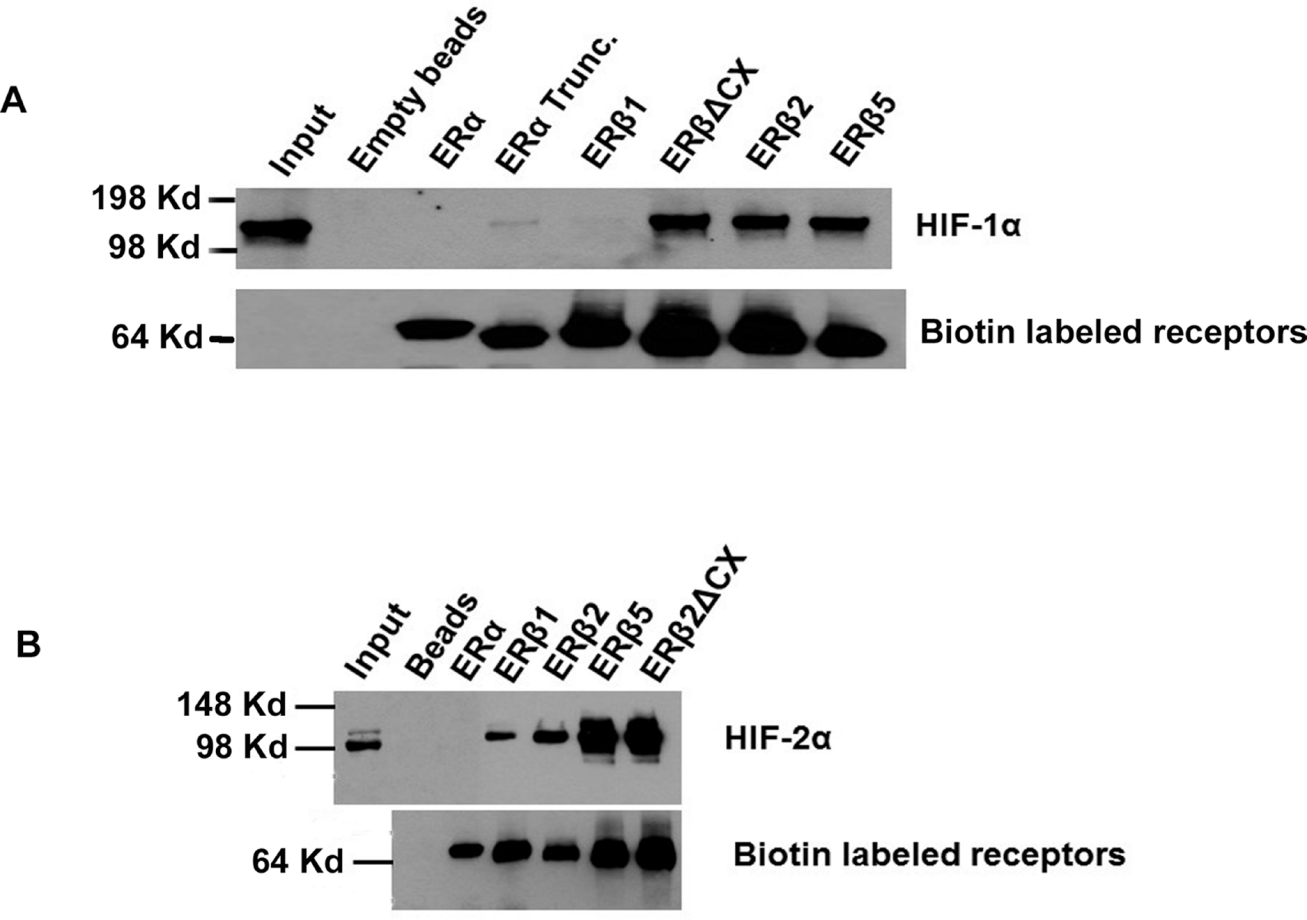
(**A**) Co-immunoprecipitations in PC3 cells for detecting HIF interactions. Transfection of PC3 cells with receptors ERα, ERα-Trunc, ERβ1, ERβΔCX (Same as ERβ2 but lacks the c-terminal spliced in peptide unique for ERβ2), ERβ2 and ERβ5 fused to biotinylation consensus, together with biotin ligase expressing plasmid BirA and expression plasmid for HIF-1α or HIF-2α. After 24 hours, cell extracts were made and biotinylated proteins were bound to streptavidin magnetic beads for 2 hours. Beads were washed three times for 10 minutes in lysis buffer, then the beads were boiled in SDS loading buffer and proteins were separated on SDS-PAGE. Co-immunoprecipitated HIF-1α protein was detected on western blot and on a separate blot biotinylated receptors were detected using Streptavidin-HRP. (**B**) The same procedure was repeated as in (**A**) exchanging HIF-1α for HIF-2α, and detecting with HIF-2α antibody.

We have shown that the ERβ variants interact with HIF-1α, however it was not known if they also could interact with HIF-2α and whether or not the C-terminal peptide of the variants was important for the interaction. We found that the variants also interacted with HIF-2α (Figure 6A and 6B) and that the small C-terminal peptide was dispensable for the interaction. Unexpectedly, we also found that ERβ1 showed some interaction with HIF-2α, while ERα did not. Regarding the HIF-1α interactions, we also used an ERα construct truncated at the corresponding amino acid as with ERβ2, to investigate if truncation of ERα could cause interaction with HIF-1α. As can be seen in Figure 6A, only a minor interaction occurred with a truncated ERα.

### 4-OH Tamoxifen inhibited the increased proliferation caused by ERβ2

**Figure 7:**
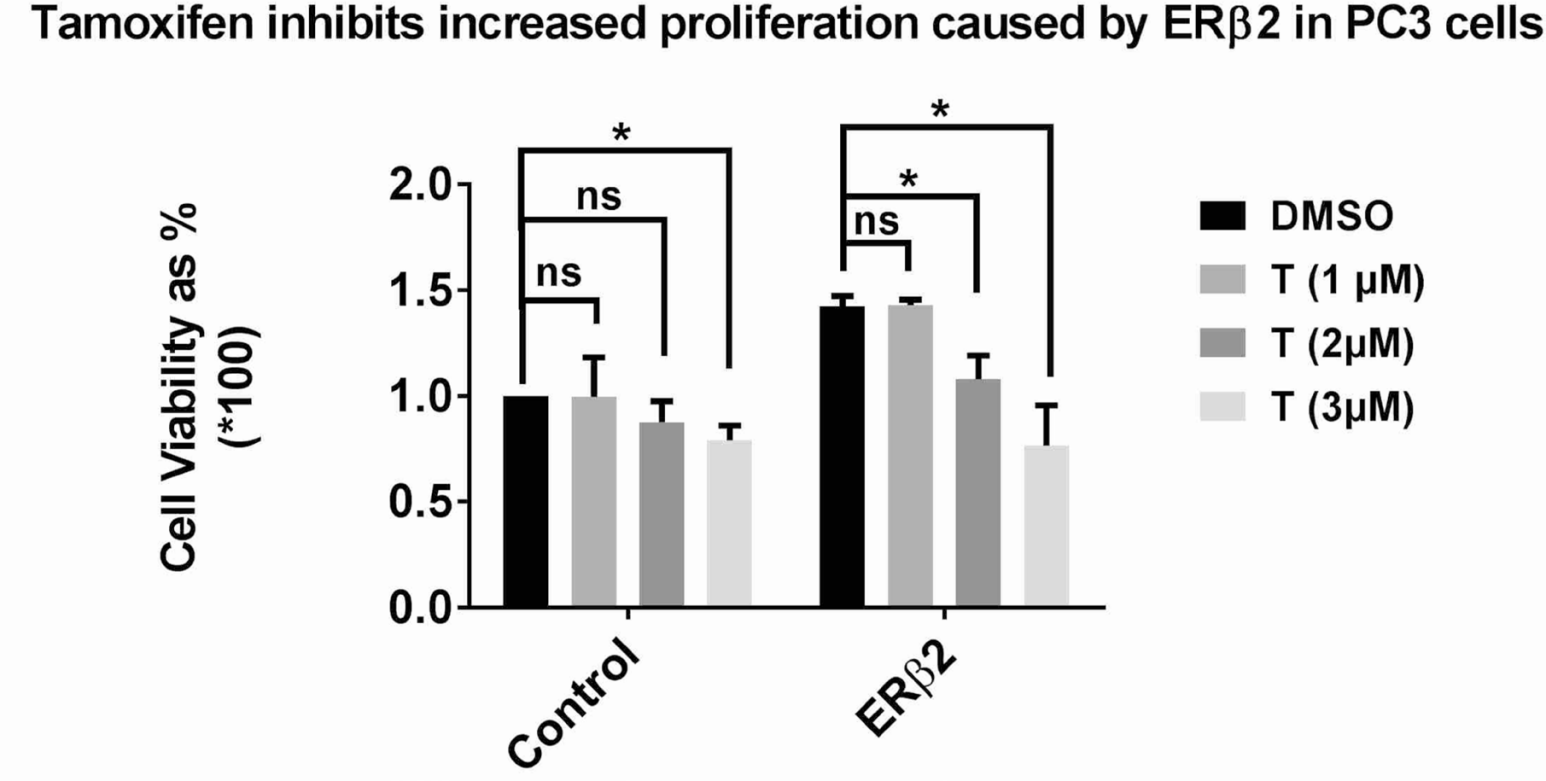
PC3 cells (control and ERβ2) plated on 96 well plate and treated with increasing doses of 4OH tamoxifen MTS assay was performed after 72 hours.

In a previous report we have shown that ERβ2 causes increased proliferation when expressed in PC3 and 22Rv1 cells [8]. Since ERβ2 has a truncated ligand-binding-domain, but the truncation is after the ligand binding pocket, there is a possibility of a residual pocket available for binding ligands. We treated PC3 cells expressing ERβ2 with 4OH-tamoxifen at increasing doses and could observe that the increased proliferation caused by ERβ2 was inhibited in a dose-dependent manner (Figure 7).

### Transcriptome analysis of prostate cancer PDX showed clustering of 9 transcripts differing between < 5 and > 5 year survival

**Figure 8:**
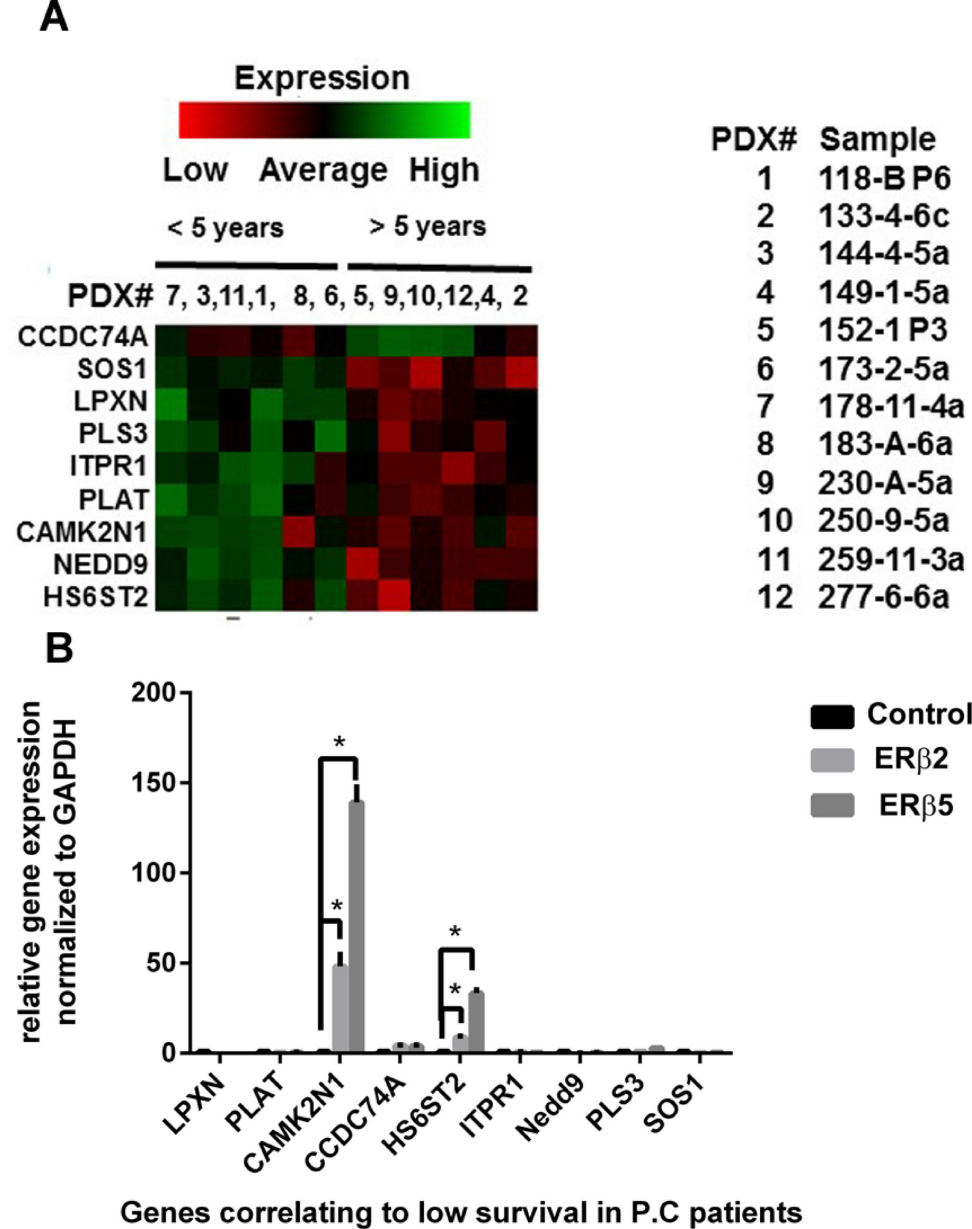
Clustering of genes related to survival in prostate cancer PDX (**A**) RNA-seq of 12 MDA PCa PDXs derived from prostate cancers with different clinical prognosis and treatments. Clustering of 9 transcripts that show different expression patterns between less than 5 year survival and longer than 5 year survival. (**B**) Graph showing regulation of the 9 transcripts by ERβ2 and ERβ5 in PC3 cells.

We performed transcriptome analysis on 12 prostate cancer PDX from the MDA PCa PDX series. ERβ transcript was found at low levels in 10 of the 12 samples, with one sample (no. 3) showing higher expression. No correlation of total ERβ expression to patient outcome was detected. However, at that stage, we did not know whether the expressed mRNA was the full length ERβ or whether it represented ERβ splice variants, which might explain the lack of correlations. By comparing the transcriptome in patients with less than 5 years-survival to those with more than 5 years survival, we found 9 transcripts whose expression was different between the two groups (Figure 8A). It was interesting to note that these transcripts did not correlate to the site of the tumor i.e. primary tumor or metastasis, or any treatment as shown in the clinical data for the MDA PCa PDXs (Supplementary Table 2). Many of these genes have been shown to have effects on EMT and metastasis [30–35]. Two of these transcripts were regulated by the ERβ variants in PC3 cells (Figure 8B).

## DISCUSSION

Our findings underline the complexity by which the ERβ gene regulates cellular events involved in cancer. In a previous report we have described the opposite effects of ERβ1 and ERβ2 in prostate cancer with respect to proliferation and apoptosis [8]. In this study, we also included ERβ5 which, in addition to ERβ2 has been shown to confer poor prognosis when expressed in prostate cancer [36]. To find similarities and differences between these ERβ isoforms, we performed RNA-seq on PC3 cells expressing ERβ2 or ERβ5 compared to control. The many mutually regulated transcripts, 7054, indicated that their mechanism of action is to a large degree overlapping; however, there were around 1000 unique transcripts for each variant allowing for some differences. One of the most upregulated mutual transcripts was cyclin D2. It has earlier been reported that cyclin D2 is critical for proliferation of glioblastoma stem cells [26]. Cyclin D2 is also indirectly regulated by hypoxia through HIF-2α, which increases the activity of c-Myc on the cyclin D2 promoter [27]. ERRβ transcript was increased by both splice variants, which is contradictory to a study showing that ERRβ has a tumor suppressive role in prostate cancer [37]. However, ERRβ was shown to be regulated by hypoxia and in addition, to interact with Oct4 to promote stem cell renewal [18, 38]. The previous study suggested that ERRβ is expressed in cells with stem cell characteristics, but not in the bulk tumor. We found that ERβ5 reduces the protein level of ERRβ from control cells, indicating a different mechanistic action between ERβ2 and ERβ5. c-kit was also strongly induced at the transcript level by both variants. However, no changes could be detected at the protein level between control and ERβ2. There was a clear reduction of c-kit in ERβ5 expressing cells. The c-kit ligand, SCF, was regulated in the same way, again indicating a different mechanism between the splice variants. Notch3 has been shown to be associated with cancer stem cells (CSC) in ovarian cancer, where its expression correlates with bad prognosis [21]. In addition, it has also been shown to be regulated by hypoxia [39]. Notch3 was shown to be inversely associated with survival in prostate cancer, indicating its increase during cancer progression [40]. RNA seq revealed increased transcripts of the Notch3 target genes, HES5 and Hey2. HES5 has been shown to increase activation of STAT3 and STAT3 promotes glioblastoma stem cell renewal [24, 41], and acts synergistically with Nanog to maintain pluripotency of embryonic stem cells [42]. In addition, we also found increased STAT3 expression by both splice variants. Other highly induced hypoxia regulated transcripts involved in stem cell proliferation were, WNT11, and the lncRNA HOTAIR. In addition, ID4, another factor highly induced by the ERβ variants has been shown to control mammary stem cells and mark breast cancers with a stem cell like phenotype [25] and when expressed in prostate cancer is associated with increased risk for distant metastases [43].

In addition to HOTAIR, the lncRNA XIST was strongly regulated. XIST has been shown to be oncogenic in non-small cell lung cancer, where it increases Bcl-2 levels by functioning like a sponge for miR-449a, which then down-regulates Bcl-2 [44]. In Glioma, XIST increases Rac-1 signaling by sequestering miR-137 [45], in colorectal cancer, it increases MAPK1 signaling by sequestering miR-132-3p [46] or by sequestering miR378, which targets MAPK1 in prostate. The last two scenarios are the most likely in our case, since we saw strong up-regulation of MAPK1 signaling (Supplementary Figure 3A–3C). Osteopontin has been shown to promote metastasis of cholangiocarcinoma through recruiting MAPK1 and mediating Ser 675 phosphorylation of β-catenin [47]. We found that ABCG2 and MDR1 were upregulated by both ERβ2 and ERβ5. Both of these MDR are regulated by hypoxia and HIF-1α expression is crucial for chemotherapy resistance in breast cancer stem cells [48]. In addition, expression of ABCG2 has been shown to correlate to cancer stem cells, explaining their unique ability to export Hoechst333342 dye out of the cells, creating a side population when using FACS to isolate cancer stem cells [49]. ABCG2 expression may correlate to worse prognosis in prostate cancer, partly by its correlation to cancer stem cells and partly by its ability to cause chemotherapy resistance. In conclusion, expression of the ERβ variants in PC3 cells induced a chemotherapy resistant, stem cell-like phenotype. Using siRNA to HIF-1α attenuated ERβ-mediated induction of ABCG2, but did not affect induction by ERβ5. Thus, there was a HIF-1α independent mechanism of induction of ABCG2. Activation of NF-κB signaling correlates to worse prognosis in many cancers, including prostate cancer [50], and leads to chemotherapy resistance. We showed that the ERβ variants’ induction of ABCG2 expression was dependent on IKKβ activity and that the IKKβ protein was increased by variant expression, indicating that NF-κB activity was important for causing chemotherapy resistance. It has been shown that ERβ1 represses HIF-1α signaling [51]. The mechanism of HIF-1α repression was later shown to be mediated by ERβ1’s direct upregulation of PHD2 which increases degradation of HIF-1α [9]. Furthermore, ERβ1 has been shown to increase chemotherapy sensitivity while the variants are decreasing the sensitivity as shown in this study. Since ERβ1 is most of the time co-expressed with its variants it is likely that the ratio of ERβ1 to its variants will determine the chemotherapy sensitivity.

RNA-seq analysis of 12 MDA PCa PDXs with varying clinical background gave us 9 transcripts that were changed between short survival (<5 years) and longer survival (>5 years). These transcripts did not correlate to any parameter such as tumor site, primary tumor vs. metastasis or treatments such as hormone therapy, chemotherapy or radiation. However, many of the transcripts had a connection to TGF-β signaling, EMT and migration, suggesting a more aggressive tumor when the transcripts are expressed. We found that two of the transcripts were strongly regulated by the variants. One was CAMK2N1, which is an inhibitor of CAMK2 and appears to be a tumor suppressor in prostate cancer [52]. However, other reports describe that its expression in prostate cancer correlates to recurrence [53], indicating a more complex behavior of this gene. The second transcript was heparin sulfate 6-O-sulfotransferase 2 (HS6ST2), which affects adhesion and migration and its expression has furthermore been correlated to poor prognosis and reduced survival in gastric cancer [32]. Interestingly, this gene is also regulated by Twist [54] and was up regulated by the variants in the RNA-seq analysis. Our findings strongly point to a function of the ERβ variants in maintaining cancer stem cells. Experiments are ongoing to localize their expression in tumors using specific antibodies and to investigate if ERβ2 and ERβ5 expressing cells also express stem cell markers. A treatment specifically targeting the variants can be an option to avoid therapy resistant cancer.

## MATERIALS AND METHODS

### Reagents and cell culture

The 22Rv1, DU145 and PC3 cell lines were obtained from the American Type Culture Collection (ATCC). 22Rv1 cells were maintained in RPMI-1640 (Invitrogen Inc., Carlsbad, CA) medium supplemented with 10% fetal bovine serum (FBS) (Sigma, St. Louis, MO), 25 mM HEPES buffer and 2 mM L-glutamine (Invitrogen Carlsbad, CA), while PC3 cells were maintained in RPMI-1640 (Invitrogen Inc., Carlsbad, CA) medium supplemented with 10% fetal bovine serum (FBS) (Sigma, St. Louis, MO). All experiments used cells below passage 30. ABCG2 inhibitor YHO-13351 was obtained from Sigma St. Louis, MO, IKKβ inhibitor IMD-0354 from R&D Systems Inc. Minneapolis, MN.

Prostate cancer Patient Derived Xenografts (PDX), (MDA PCaPDXs series) used in this work were developed in the laboratory of Dr. Navone at the “Prostate Cancer PDX”-the department of Genitourinary Medical Oncology-MD Anderson Cancer Center, Houston, Texas and the David H. Koch Center for Applied Research of Genitourinary Cancers, at the same location. PDXs were established following previously described procedures [15] and propagated as subcutaneous xenografts in 6- to 8-week-old male CB17 SCID mice (Charles River Laboratories, Wilmington, MA). Patients who donated the tissue from which the xenografts were developed (*n* = 12) provided written informed consent prior to sample acquisition, and samples were manipulated and distributed according to protocols approved by the Institutional Review Board of The University of Texas M.D. Anderson Cancer Center. The PDX development used in this study is approved under protocol 00001091-RN01.

All animal experiments were conducted in accordance with the standards of the Institutional Animal Care and Use Committee of MD Anderson. The study is exempt from Institutional Review Board approval at University of Houston on the basis of non-identifiable patients.

### Construction of an inducible system for ERβ2 and ERβ5 in PC3 cells

A transposon-based tet-off system mediating doxycycline-regulated expression of ERβ2 and ERβ5 was used to stably transfect 22Rv1, DU145 and PC3 prostate cancer cells. These cell lines are described elsewhere [8]. In all experiments the cells were grown in the absence of doxycycline allowing full expression of ERβ2 or ERβ5. Doxycycline was only used to turn off expression during expansion of cells to prevent phenotypic changes from long term expression of the variants.

### RNA-seq and bioinformatic analyses of PC3 cells expressing ERβ2 or ERβ5

RNA was prepared using the Qiagen RNA-easy kit. For library preparation, Truseq stranded mRNA (Illumina) was used. The sequencing was performed on Illumina Hiseq 2000 with 50 bp single read. The reads were first mapped to the latest UCSC transcript set using Bowtie2 version 2.1.0 [55] and the gene expression level was estimated using RSEM v1.2.15 [56], TMM (trimmed mean of *M*-values) was used to normalize the gene expression. Differentially expressed genes were identified using the edgeR program [57]. Genes showing altered expression with *p* < 0.05 and more than 1.5 fold changes were considered differentially expressed. The pathway and network analysis was performed using Ingenuity Pathway Analysis (IPA). IPA computes a score for each network according to the fit of the set of supplied focus genes. These scores indicate the likelihood of focus genes to belong to a network versus those obtained by chance. A score > 2 indicates a <= 99% confidence that a focus gene network was not generated by chance alone. The canonical pathways generated by IPA are the most significant for the uploaded data set. Fischer’s exact test with FDR option was used to calculate the significance of the canonical pathway.

### RNA-seq library preparation and sequencing of PDX samples

Extracted RNA samples underwent quality control (QC) assessment using the RNA Nano 6000 chip on Bioanalyzer 2100 (Agilent) and were quantified with Qubit Fluorometer (Thermo Fisher). The RNA libraries were prepared and sequenced at University of HoustonSeq-N-Edit Core per standard protocols. Total RNA libraries were prepared with Ovatio Universal RNA-Seq System (NuGen) using 100 ng input RNA. The size selection for libraries was performed using SPRIA Select magnetic beads (Beckman Coulter) and purity of the libraries was analyzed using the High Sensitivity DNA chip on Bioanalyzer 2100 (Agilent). The prepared libraries were pooled and sequenced using Illumina NextSeq 500, generating 10–20 million 2 × 76 bp paired-end reads per sample.

### Transcriptome analysis

The RNA-seq raw fastq data were processed with RNA-Seq Alignment app within the Illumina Base Space app suite (https://www.basespace.illumina.com): the adaptors were trimmed and reads were mapped to hg19 human reference genome using the STARaligner (Dobin *et al.*, 2013) to generate the BAM files, and FPKM estimation of reference genes and transcripts were performed using Cufflinks 2 (Trapnell *et al.*, 2013). Based on this gene count matrix, we used “DESeq2” package (Love *et al.*, 2014) to identify differentially expressed genes between patients surviving less than 5 years vs. patients surviving over 5 years. The significance level of FDR adjusted *p* value of 0.05 was used to identify differentially expressed genes. The RNA-seq data is available in NCBI’s Gene Expression Omnibus through accession number GSE118449.

### Chemotherapy treatment and MTS assay

PC3 cells expressing GFP (control), ERβ2 and ERβ5 were seeded in a 96 well plate at a cell density of 1.5 × 10^4^ cells/well in 0.5% FBS, RPMI media. Chemotherapy (docetaxel Sigma, St. Louis, MO) at different nM concentrations was administered to the cells for 48 hours and 20 μl of MTS reagent was added to each well. Absorbance was measured at 490 nm after 30 minutes of adding the MTS reagent, as recommended by the company protocol (Biovision). Absorbance measurements were used to calculate treatment effects on each clone compared to the untreated control and corresponding graphs with standard deviation from the mean were plotted using Graph pad Prism software.

### Protein extract preparation

To prepare whole-cell extracts, cells were washed twice with PBS, lysed in 10 times packed cell volume of lysis buffer [0.1% Nonidet P-40, 250 mM KCl, 5 mM Hepes, pH 7.9,10% (vol/vol) glycerol, 4 mM NaF, 4 mM sodium orthovanadate, 0.2 mM EDTA, 0.2 mM EGTA, 1 mM dithiothreitol, 1 mM phenylmethylsulfonyl fluoride, protease inhibitor cocktail, PhosStop (Roche, Indianapolis, IN)] for 15 minutes on ice and then centrifuged at 14,000 × g for 10 minutes.

### Western blotting

Thirty µg of protein were loaded on an SDS-PAGE 10% Bis-Tris gel with Tris running buffer and transferred to a nitrocellulose membrane after electrophoresis separation. Membranes were blocked with 5% non-fat powdered milk in TBS buffer containing 0.1% Tween 20 and probed with antibodies. Primary antibodies were used at 1:500-1000 dilutions, and secondary antibodies were used at 1:10,000 dilutions. Antibodies and source of antibodies are given in Supplementary Table 3.

### RNA extraction and real-time PCR

RNA extraction was performed with Qiagen mRNA extraction kit according to standard protocol. cDNA was synthesized from 1 μg of total RNA with First Strand System according to standard protocol (Invitrogen Inc. NY). Real-time PCR was performed with SYBR Green I dye master mix (Applied Biosystems Foster City, CA). Primers were obtained from (Integrated DNA Technologies, Inc. Coralville, IA) and qPCR reactions were performed with a 7500 Fast Real-Time PCR System (Applied Biosystems) using optimized conditions for SYBR Green I dye system: 50° C for 2 minutes, 95° C for 10 minutes, followed by 40–50 cycles at 95° C for 15 seconds and 60° C for 50 seconds. Optimum primer concentration was determined in preliminary experiments, and amplification specificity confirmed by dissociation curve analysis. The sequences of primers used are given in Supplementary Table 3.

### Pull down from PC3 cells

PC3 cells were transfected with 3 µg BirA (biotin ligase) expression plasmid, 3 μg plasmid containing biotinylation consensus tagged receptors B7TEV-ERα, B7TEV- ERα trunc. (ERα truncated at the corresponding amino acid in the variants preceding the variant specific C-terminal peptide), B7TEV-ERβ1, B7TEV-ERβ2 and B7TEV-ERβ5 together with 3 μg pcDNA3 HA-HIF-1α “Addgene plasmid 18949” or pcDNA3 HA-HIF-2α “Addgene plasmid 18950” in a 100 mm tissue culture plate. After 48 hours, cells were scraped, pelleted and lysed in 300 μl NETN (20 mM Tris (pH = 8.0), 100 mM NaCl, 1 mM EDTA, 0.5% Nonidet P-40), briefly sonicated and centrifuged to remove debris. 10 µl streptavidin magnetic beads (Pierce Rockford, IL) were washed in NETN buffer and incubated with 300 µl cellular extract for 2 hours in the cold room. Beads were washed (3 × 10 minutes rotation) with 300 μl NETN and boiled with 20 μl of 2× sample loading buffer [65.8 mM Tris HCl (pH 6.8), with 2.1% SDS, 26.3% (w/v) glycerol, and 0.01% bromophenol blue] and subjected to SDS-PAGE and transferred to nitrocellulose membrane for Western blot. The blot was probed with HIF-1α or HIF-2α antibody (Santa Cruz Dallas, TX) 1:1000 dilution and secondary antibody 1:10,000 dilution. Biotinylated receptors where visualized using streptavidin HRP 1:10,000 dilution in PBS on a membrane blocked with 1% BSA, washing and imaging were performed using ECL.

### Hypoxia assay

PC3 cells expressing GFP (control), ERβ2 and ERβ5 were seeded in 6 well plates in duplicates at 50% cell density in 10% FBS, RPMI. Cells were exposed to hypoxia (1% O_2_) in a hypoxia chamber for 3 h and cells were harvested for protein extracts in RIPA buffer. An identical plate of 6 wells seeded with PC3 clones was kept in normoxia (21% O_2_) to use as a control. Protein extracts were made from the normoxia plate too. Both normoxia and hypoxia extracts were run on 10% SDS-PAGE gels and transferred to nitrocellulose membranes to probe with antibodies against HIF-1α, HIF-2α, ERβ1 and β-actin.

### Knockdown studies using siRNA technology

PC3 cells expressing GFP (control), ERβ2 and ERβ5 were seeded in a 24 well plate in duplicates at 30% cell density in 10% FBS, RPMI. Cells were transfected with 20 pM siRNA for luciferase, (AM4629 Thermo Fisher), HIF-1α (s6541 Thermo Fisher) or HIF-2α (s4698 Thermo Fisher) with lipofectamine 2000 in a 24 well plate, incubated for 48–72 hours, after which RNA or protein extracts were prepared and analyzed.

### Statistics

The values are expressed as the mean with 95% confidence intervals. An unpaired, two-tailed *t*-test was used to compare the differences between the control group and each experimental group. The significance is presented as ^*^p < 0.05, ^**^p < 0.005, and ^***^p < 0.001, and non-significant differences are presented as NS.

## SUPPLEMENTARY MATERIALS FIGURES AND TABLES















## Abbreviations

LBD: ligand binding domain
ERE: estrogen response element
ADT: androgen deprivation therapy
CRPC: castration resistant prostate cancer
SERM: selective estrogen receptor modulators
MDR: multidrug resistance
PDX: patient derived xenograft
MDA: MD Anderson
EMT: epithelial mesenchymal transition
