# Supplementary material for: The estrogen receptor variants β2 and β5 induce stem cell characteristics and chemotherapy resistance in prostate cancer through activation of hypoxic signaling
Source: Oncotarget. 2018 Nov 20;9(91):36273–88. doi: 10.18632/oncotarget.26345 (PMC6284737; doi:10.18632/oncotarget.26345)
Supplement: Supplementary file 1 [file oncotarget-09-36273-s001.pdf]

The estrogen receptor variants β2 and β5 induce stem cell characteristics and chemotherapy resistance in prostate cancer through activation of hypoxic signaling

SUPPLEMENTARY MATERIALS

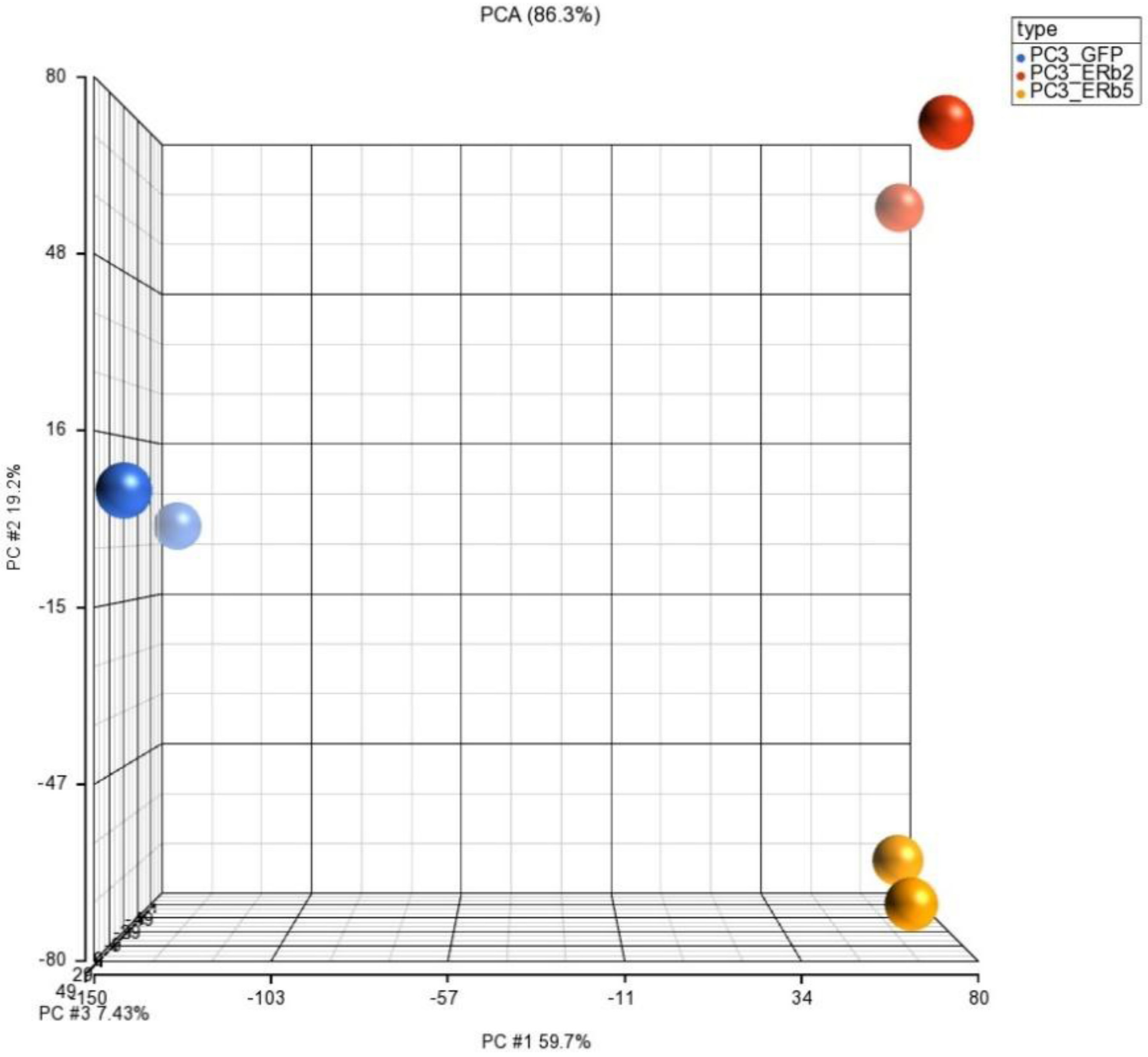

Supplementary Figure 1: Principal component analysis.

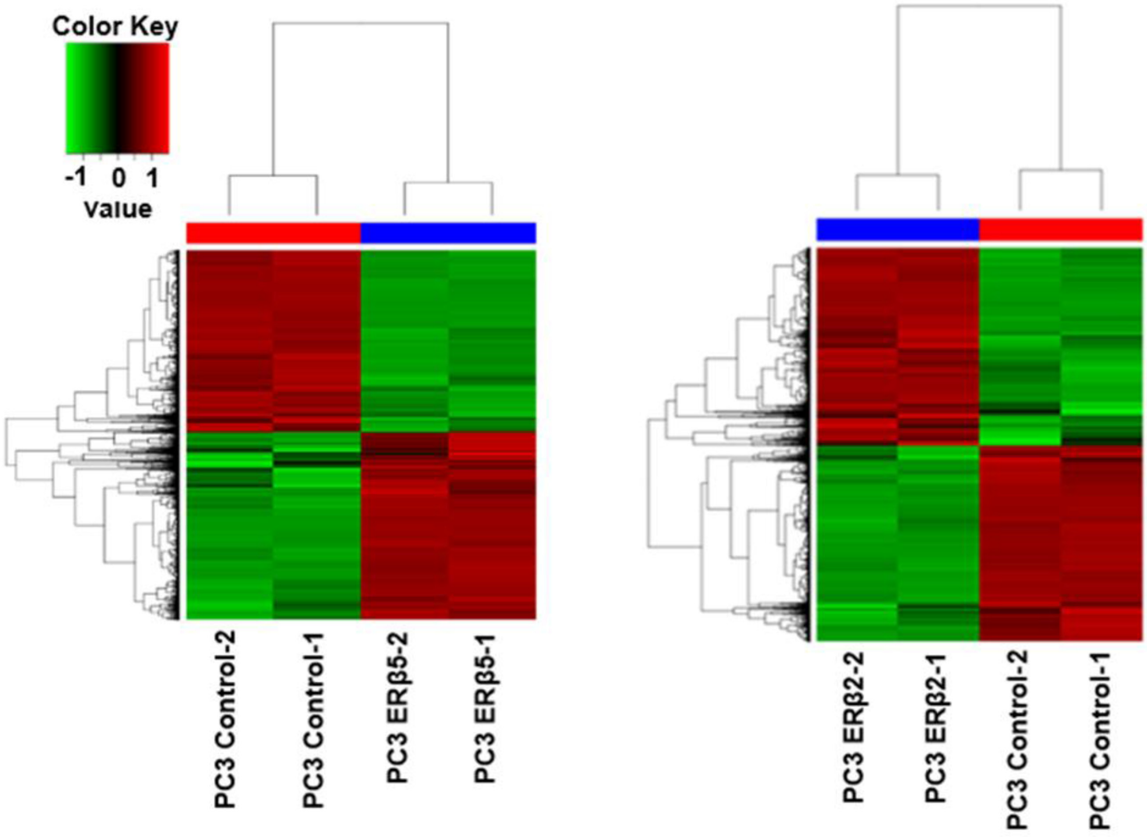

Supplementary Figure 2: Heatmap showing high similarity between repeat samples and big difference between controls and ERβ2/5 expressing cells.

A

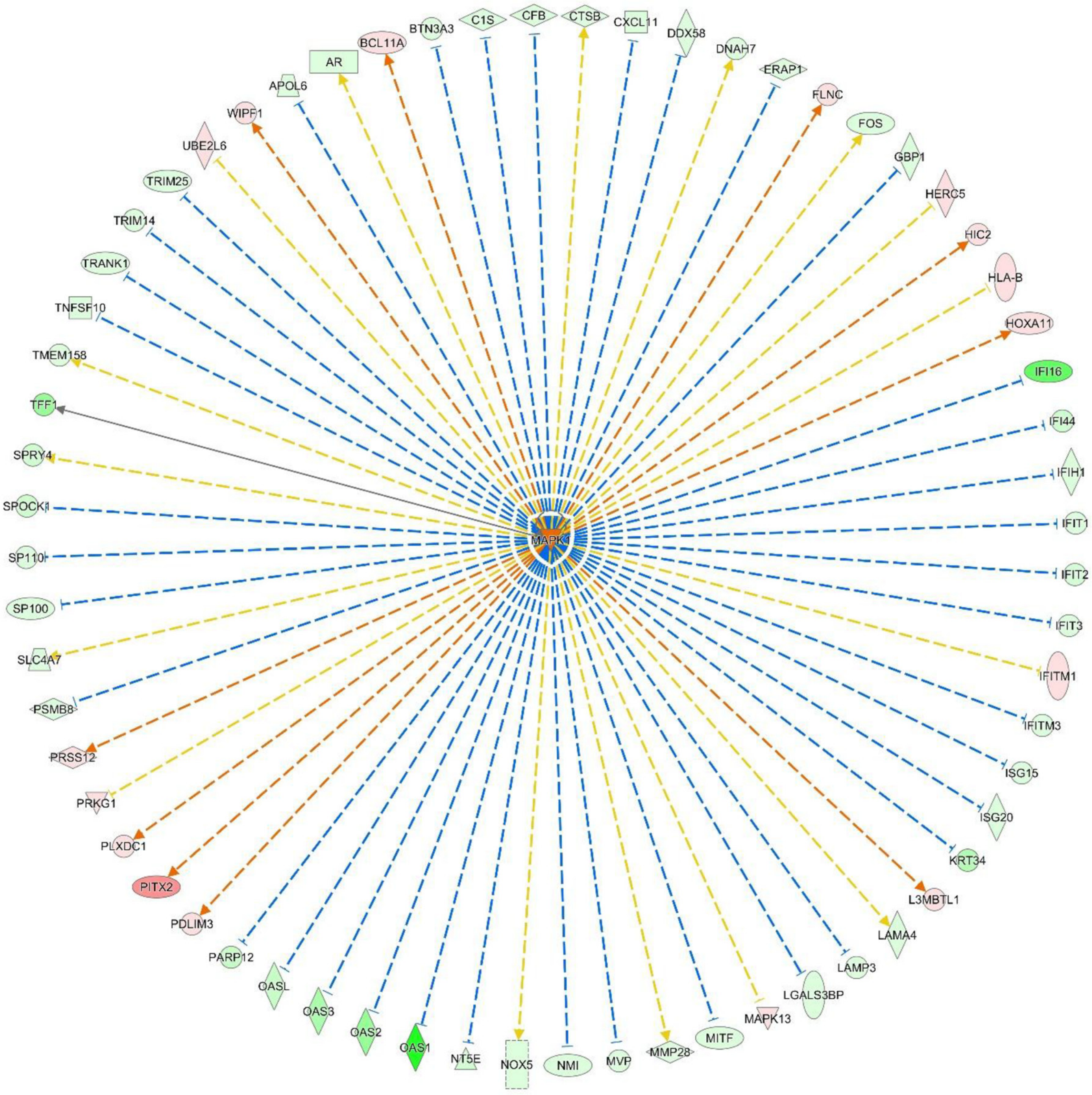
MAPK1
BTN3A3
C1S
CFB
CTSB
CXCL11
DDX58
DNAH7
ERAP1
FLNC
FOS
GBP1
HERC5
HIC2
HLA-B
HOXA11
IFI16
IFI44
IFIH1
IFIT1
IFIT2
IFIT3
IFITM1
IFITM3
ISG15
ISG20
KRT34
L3MBTL1
LAMA4
LAMP3
LGALS3BP
MAPK13
MITF
MMP28
MVP
NMI
NOX5
NT5E
OAS1
OAS2
OAS3
OASL
PARP12
PDLIM3
PITX2
PLXDC1
PRKG1
PRSS12
PSMB8
SLC4A7
SP100
SP110
SPOCK1
SPRY4
TFF1
TMEM158
TNFSF10
TRANK1
TRIM14
TRIM25
UBE2L6
WIPF1
APOL6
AR
BCL11A

B

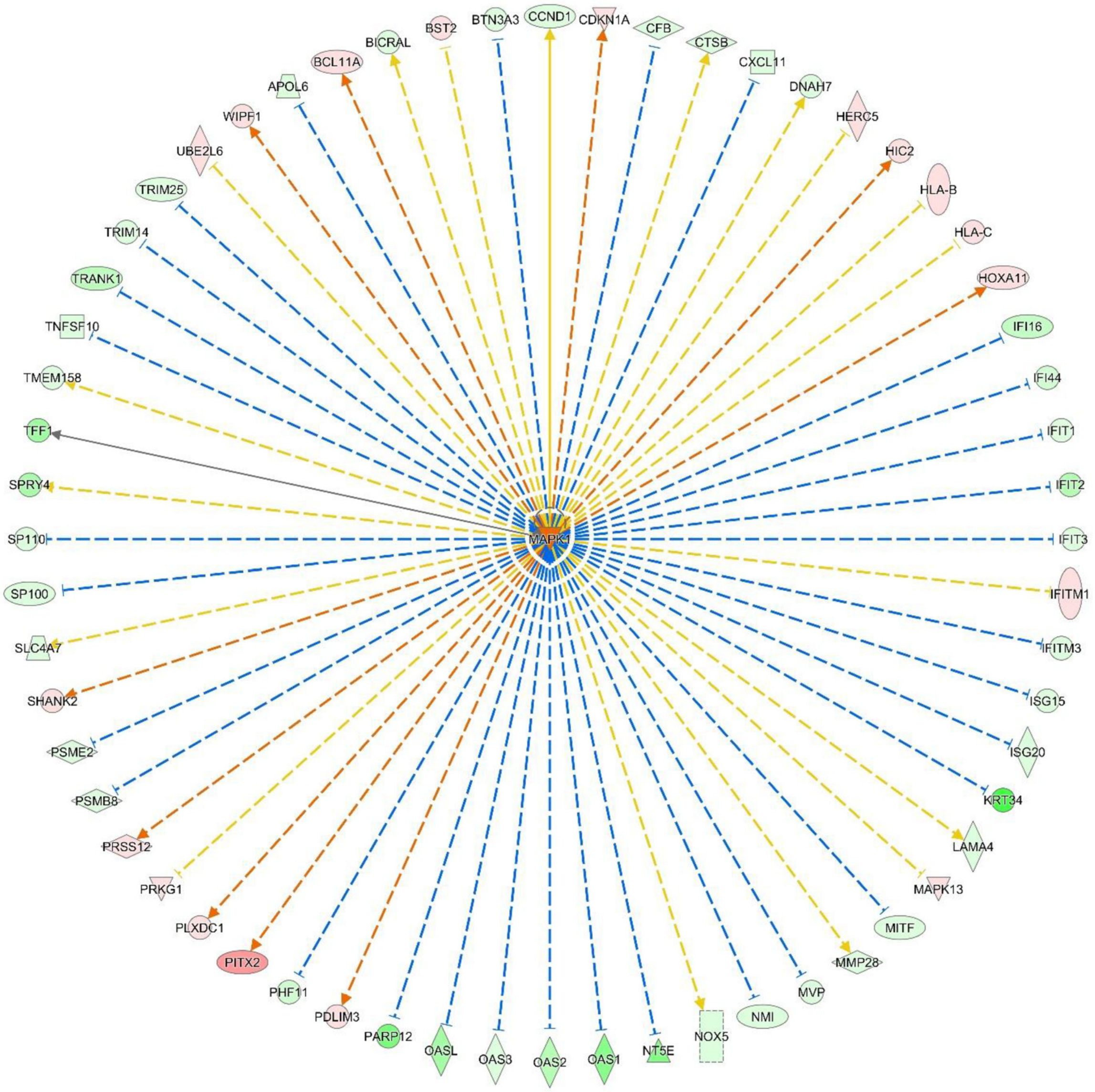
MAPK1
CCND1
CDKN1A
CFB
CTSB
CXCL11
DNAH7
HERC5
HIC2
HLA-B
HLA-C
HOXA11
IFI16
IFI44
IFIT1
IFIT2
IFIT3
IFITM1
IFITM3
ISG15
ISG20
KRT34
LAMA4
MAPK13
MITF
MMP28
MVP
NMI
NOX5
NT5E
OAS1
OAS2
OAS3
OASL
PARP12
PDLIM3
PHF11
PITX2
PLXDC1
PRKG1
PRSS12
PSMB8
PSME2
SHANK2
SLC4A7
SP100
SP110
SPRY4
TFF1
TMEM158
TNFSF10
TRANK1
TRIM14
TRIM25
UBE2L6
WIPF1
APOL6
BCL11A
BICRAL
BST2
BTN3A3

C

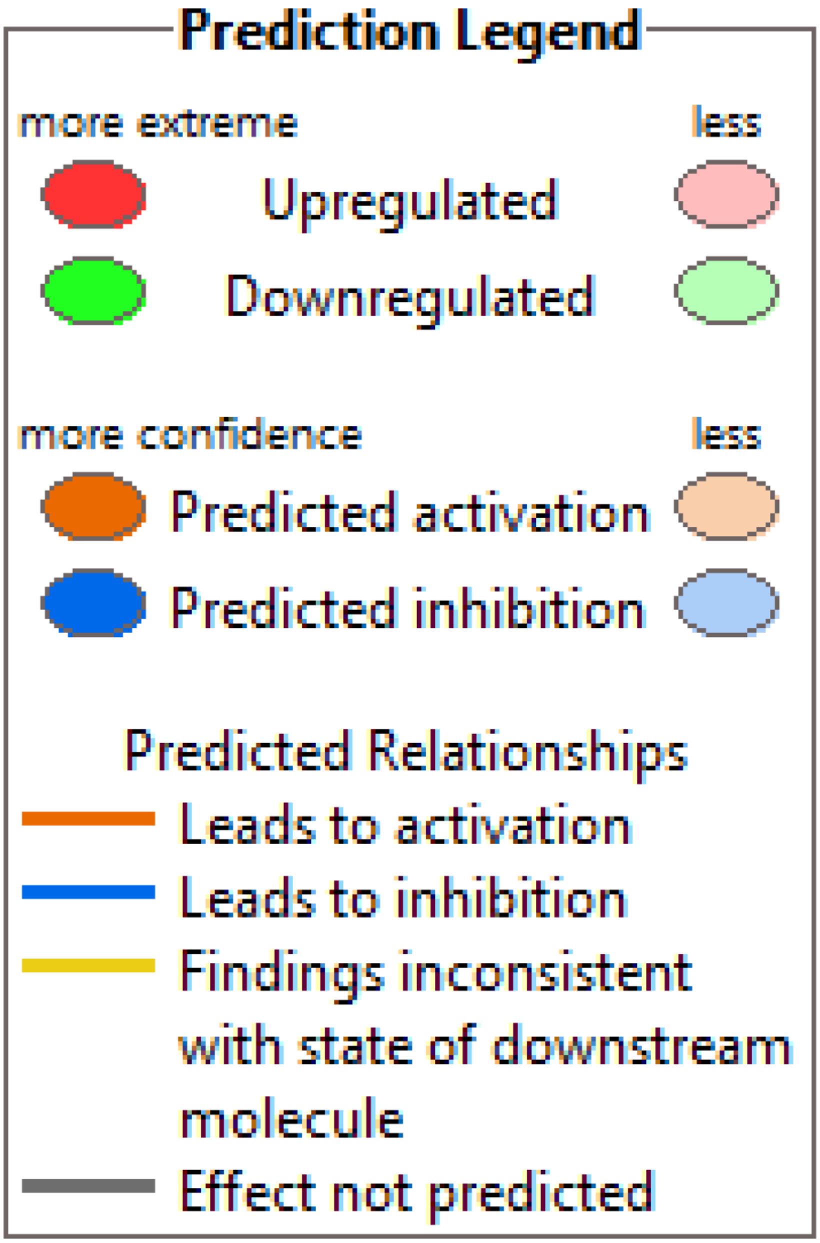

Supplementary Figure 3: (**A**) ERβ2 increases MAPK1 signaling. (**B**) ERβ5 increases MAPK1 signaling. (**C**) Color codes for (A) and (B).

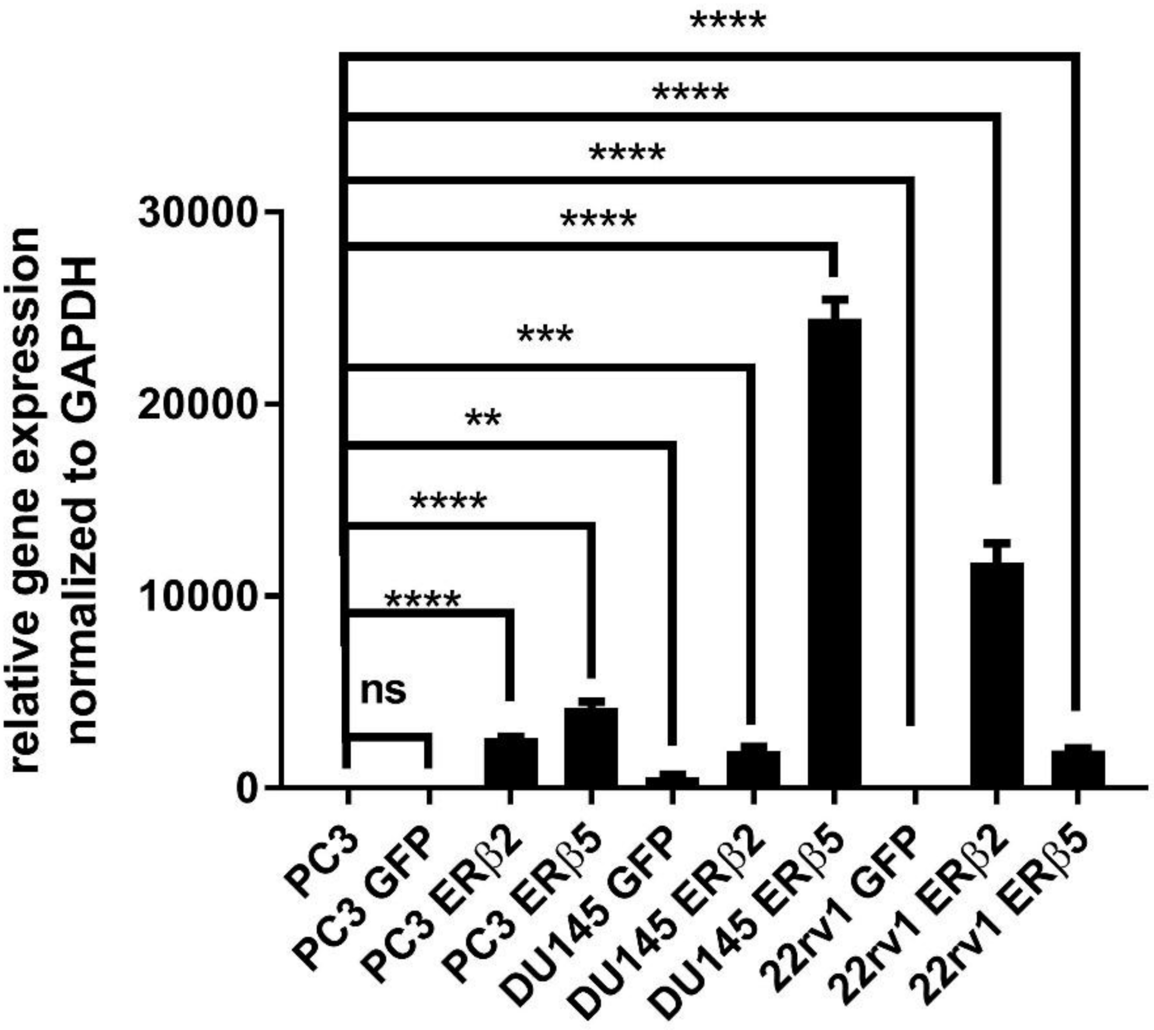

Supplementary Figure 4: ERβ2 and ERβ5 expression in three different prostate cancer stable cell lines.

Supplementary Table 1A: Selected mutually up regulated transcripts. See Supplementary_Table_1A

Supplementary Table 1B: Top 30 mutually down regulated transcripts. See Supplementary_Table_1B

Supplementary Table 1C: Top 30 up regulated transcripts for ERβ2. See Supplementary_Table_1C

Supplementary Table 1D: Top 30 down regulated transcripts for ERβ2. See Supplementary_Table_1D

Supplementary Table 1E: Top 30 up regulated transcripts for ERβ5. See Supplementary_Table_1E

Supplementary Table 1F: Top 30 down regulated transcripts for ERβ5. See Supplementary_Table_1F

Supplementary Table 2: ERβ expression in prostate cancer PDX from the MDA PCa PDX series (UT MD Anderson Cancer Center, Texas)

	PDX name: MDA PCa	Total ERβ FPKM	Diagnosis & Tumor site	Grade	Therapy	Clinical state	Survival from diagnosis (years)
1	118-b	0.722384	Metastatic adenocarcinoma, bone	N/A	HT, CT, RT	CRPC	2.9
2	133–4	0.232289	Metastatic carcinoma, bone	N/A	HT, CT, RT	CRPC	8.6
3	144–4	3.873795	Adenocarcinoma with NE differentiation and small cell carcinoma, prostate	N/A	HT, CT, RT	CRPC	4.8
4	149–1	0.714604	Adenocarcinoma, ureter	4+5	HT, CT, RT	CRPC	6.9
5	152–1	0	Metastatic adenocarcinoma (ductal), brain	N/A	HT, RT	CRPC	11.4
6	173–2	0.037032	Adenocarcinoma, prostate	3+4	None	N	4
7	178–11	0.389715	Adenocarcinoma, prostate	4+5	HT	Ar	3.4
8	183-A	0	Metastatic carcinoma, bone	N/A	None	N	2.3
9	230-A	0.282839	Metastatic Adenocarcinoma, chest wall	N/A	HT, CT, RT	CRPC	10.7
10	250-9	0.123058	Metastatic Adenocarcinoma, adrenal gland	N/A	HT	Ar	5.7
11	259–11	0	Adenocarcinoma, prostate	4+5	None	N	4
12	277–6	0.105902	Metastatic small cell carcinoma, lymph node	N/A	HT, CT, RT	CRPC	9.9

Abbreviations: HT = Hormone Therapy, CT = Chemotherapy, RT = Radiotherapy, CRPC = Castration-resistant prostate cancer, Ar = Androgen responsive, N = Naïve, FPKM = Fragments per Kilobase of Exon per Million Fragments Mapped.

Supplementary Table 3: List of antibodies and source and list of primers used in the study

Antibodies	Source	Order number
Cyclin D2	Santa Cruz	Sc-593
Notch3	Cell Signaling Technology	D11B8
WNT11	AbCam	Ab31962
ERRβ	AbCam	Ab104804
HIF-1α	Santa Cruz	Sc-10790
HIF-2α	Santa Cruz	Sc-46691
SCF/Kit	AbCam	Ab52603
ABCG2	Santa Cruz	Sc-25822
IKKβ	Cell Signaling Technology	D30C6
ERβ	GeneTex	GTX70174 (14C8)
β-actin	Sigma	A-1978
ID4	Santa Cruz	Sc-365656

Primer	Forward	Reverse
ABCG2	5′AGGTGCAAGGAAAGATCCAA	5′TCCAGACACACCACGGATAA
GAPDH	5′TGACAACTTTGGTATCGTGG	5′AGGCAGGGATGATGTTCTGGA
MDR1	5′AGGAAGCCAATGCCTATGACT	5′CAACTGGGCCCCTCTCTCTC
LPXN	5′ACGCTCCACCCTTCAGGACA	5′GACATTGAGCTCCTGGATATTGG
PLAT	5′CCGGCTACGGCAAGCA	5′AGCGGCTGGATGGGTACA
CCDC74A	5′TCTAATTCAGGCAAGGCCAGG	5′AGTAGGGGCTCCTCTTCCAG
HS6ST2	5′GAAGCAGAACTCAGGCAAGG	5′CCAATGAAGGAAGCAGGATGT
ITPR1	5′CTGCCACCAGTTCAAAAGCC	5′CCACCTCTGCTGCCAAGTAA
NEDD9	5′GAGCTGGATGGATGACTACGA	5′AGCTCTTTCTGTTGCCTCTCA
PLS3	5′ACAGAAGAAGACAAGCACAATAACG	5′CCCATCAAACAGGCAAACACG
SOS1	5′GACCAAGCGGGTTGTTATTGA	5′TGCCTTGTCGGTCATATTTTTCA
CAMK2N1	5′GACCAAGCGGGTTGTTATTGA	5′TGCCTTGTCGGTCATATTTTTCA
HOTAIR	5′GGCGGATGCAAGTTAATAAAA	5′TACGCCTGAGTGTTCACGAG
XIST	5′GCATAACTCGGCTTAGGGCT	5′TCCTCTGCCTGACCTGCTAT

Supplementary Table 4: A list of ERβ2 regulated transcripts. See Supplementary_Table_4

Supplementary Table 5: A list of ERβ5 regulated transcripts. See Supplementary_Table_5

Supplementary Table 6: A list of mutual regulated transcripts. See Supplementary_Table_6

Supplementary Table 7: A list of unique ERβ2 regulated transcripts. See Supplementary_Table_7

Supplementary Table 8: A list of unique ERβ5 regulated transcripts. See Supplementary_Table_8
