# Supplementary material for: The estrogen receptor variants β2 and β5 induce stem cell characteristics and chemotherapy resistance in prostate cancer through activation of hypoxic signaling
Source: Oncotarget. 2018 Nov 20;9(91):36273–88. doi: 10.18632/oncotarget.26345 (PMC6284737; doi:10.18632/oncotarget.26345)
Supplement: Supplementary file 2 [file oncotarget-09-36273-s002.docx]

**Supplementary Table 1A:­ Selected mutually up regulated transcripts**

| Gene_ID | NAME | Foldchange Control vs ERβ2 | Foldchange Control vs ERβ5 |
| --- | --- | --- | --- |
| CCND2 | cyclin D2 | 14210 | 11585 |
| SALL2 | spalt-like transcription factor 2 | 10754 | 6080 |
| DAPK1 | death-associated protein kinase 1 | 8977 | 7287 |
| ID4 | inhibitor of DNA binding 4, dominant negative helix-loop-helix protein | 7523 | 8389 |
| VANGL2 | VANGL planar cell polarity protein 2 | 7201 | 4924 |
| LAMA1 | laminin, alpha 1 | 6649 | 2403 |
| IGFBP2 | insulin-like growth factor binding protein 2, 36kDa | 5657 | 4990 |
| XIST | X inactive specific transcript (non-protein coding) | 5509 | 5581 |
| ALX4 | ALX homeobox 4 | 4826 | 5866 |
| FXYD6 | FXYD domain containing ion transport regulator 6 | 4779 | 4062 |
| MSI1 | musashi RNA-binding protein 1 | 4478 | 5189 |
| KIT | v-kit Hardy-Zuckerman 4 feline sarcoma viral oncogene homolog | 3338 | 3270 |
| ROR2 | receptor tyrosine kinase-like orphan receptor 2 | 2946 | 1992 |
| ABCB1 | ATP-binding cassette, sub-family B (MDR/TAP), member 1 | 2708 | 3332 |
| MAFB | v-maf avian musculoaponeurotic fibrosarcoma oncogene homolog B | 2530 | 1630 |
| FOXF1 | forkhead box F1 | 1969 | 2191 |
| FGFR3 | fibroblast growth factor receptor 3 | 1473 | 1339 |
| ESRRB | estrogen-related receptor beta | 1168 | 311 |
| STAT5B | signal transducer and activator of transcription 5B | 1027 | 1301 |
| EN2 | engrailed homeobox 2 | 967 | 747 |
| WNT11 | wingless-type MMTV integration site family, member 11 | 786 | 992 |
| SOX5 | SRY (sex determining region Y)-box 5 | 675 | 525 |
| HES5 | hes family bHLH transcription factor 5 | 653 | 782 |
| SIM1 | single-minded family bHLH transcription factor 1 | 621 | 605 |
| KLF15 | Kruppel-like factor 15 | 585 | 539 |
| PDX1 | pancreatic and duodenal homeobox 1 | 371 | 511 |
| HEYL | hes-related family bHLH transcription factor with YRPW motif-like | 359 | 402 |
| NOTCH3 | notch 3 | 293 | 139 |
| HS6ST2 | heparan sulfate 6-O-sulfotransferase 2 | 288 | 391 |
| HOTAIR | HOX transcript antisense RNA | 199 | 175 |
| TWIST2 | twist family bHLH transcription factor 2 | 138 | 230 |
| WNT5B | wingless-type MMTV integration site family, member 5B | 121 | 79 |
| SOX6 | SRY (sex determining region Y)-box 6 | 96 | 77 |
| MMP2 | matrix metallopeptidase 2 | 91 | 128 |
| GLI2 | GLI family zinc finger 2 | 90 | 76 |
| CAMK2N1 | calcium/calmodulin-dependent protein kinase II inhibitor 1 | 88 | 184 |
| STAT3 | signal transducer and activator of transcription 3 (acute-phase response factor) | 86 | 96 |
| FGFR2 | fibroblast growth factor receptor 2 | 55 | 65 |
| ABCG2 | ATP-binding cassette, sub-family G (WHITE), member 2 (Junior blood group) | 51 | 41 |
| RET | ret proto-oncogene | 50 | 36 |
| BMP7 | bone morphogenetic protein 7 | 45 | 65 |
| GREB1 | growth regulation by estrogen in breast cancer 1 | 38 | 17 |
| ERBB4 | erb-b2 receptor tyrosine kinase 4 | 38 | 23 |
| SNAI3 | snail family zinc finger 3 | 23 | 22 |
| AKT3 | v-akt murine thymoma viral oncogene homolog 3 | 18 | 16 |
| HEY2 | hes-related family bHLH transcription factor with YRPW motif 2 | 18 | 26 |
| ID2 | inhibitor of DNA binding 2, dominant negative helix-loop-helix protein | 15 | 12 |
| DACH1 | dachshund family transcription factor 1 | 13 | 9 |
| IGF1R | insulin-like growth factor 1 receptor | 13 | 11 |
| CXCR4 | chemokine (C-X-C motif) receptor 4 | 12 | 11 |
| SMAD7 | SMAD family member 7 | 12 | 14 |

**Supplementary Table 1B: Top 30 mutually down regulated transcripts**

| GeneID | NAME | Foldchange  Control vs ERβ2 | Foldchange  Contro;vs ERβ5 |
| --- | --- | --- | --- |
| TM4SF19 | transmembrane 4 L six family member 19 | -1794 | -41 |
| ARHGEF35 | Rho guanine nucleotide exchange factor (GEF) 35 | -1608 | -66 |
| SLC35 | solute carrier family 35,member F33 | -1283 | -17 |
| PCDHAC2 | protocadherin alpha subfamily C, 2 | -1213 | -74 |
| CACNA1A | calcium channel, voltage-dependent, P/Q type, alpha 1A subunit | -1131 | -68 |
| KRT86 | keratin 86, type II | -1122 | -55 |
| SLC22A3 | solute carrier family 22 (organic cation transporter), member 3 | -1036 | -62 |
| SPINK5 | serine peptidase inhibitor, Kazal type 5 | -884 | -11 |
| ONECUT3 | one cut homeobox 3 | -884 | -70 |
| ABHD14A-ACY1 | ABHD14A-ACY1 readthrough (NMD candidate) | -738 | -8 |
| DYX1C1-CCPG1 | DYX1C1-CCPG1 readthrough (NMD candidate) | -733 | -733 |
| RP1 | retinitis pigmentosa 1 (autosomal dominant) | -732 | -732 |
| SIRPB1 | signal-regulatory protein beta 1 | -714 | -43 |
| MARC4 | membrane-associated ring finger (C3HC4) 4, E3 ubiquitin protein ligase | -646 | -74 |
| SUSD3 | sushi domain containing 3 | -629 | -129 |
| PCDHA13 | protocadherin alpha 13 | -575 | -66 |
| OAS1 | 2'-5'-oligoadenylate synthetase 1, 40/46kDa | -566 | -67 |
| LOC100506860 | NA | -561 | -64 |
| MGMT | O-6-methylguanine-DNA methyltransferase | -548 | -112 |
| GJB3 | gap junction protein, beta 3, 31kDa | -504 | -74 |
| SDR16C5 | NA | -490 | -17 |
| ZNF334 | zinc finger protein 334 | -481 | -55 |
| FLG | filaggrin | -480 | -480 |
| MB21D1 | Mab-21 domain containing 1 | -476 | -70 |
| ARHGEF34P | Rho guanine nucleotide exchange factor (GEF) 34, pseudogene | -476 | -476 |
| C19orf33 | chromosome 19 open reading frame 33 | -468 | -91 |
| CTHRC1 | collagen triple helix repeat containing 1 | -453 | -84 |
| PLEK2 | pleckstrin 2 | -444 | -82 |
| SCEL | sciellin | -428 | -2022 |
| FEZF1-AS1 | FEZF1 antisense RNA 1 | -391 | -24 |

**Supplementary Table 1C: Top 30 up regulated transcripts for ERβ2**

| Gene_ID | NAME | Foldchange |
| --- | --- | --- |
| BHLHE22 | basic helix-loop-helix family, member e22 | 1582 |
| MAGEA2B | melanoma antigen family A2B | 1518 |
| CHODL | chondrolectin | 597 |
| PRR35 | proline rich 35 | 521 |
| KISS1R | KISS1 receptor | 520 |
| TCEAL7 | transcription elongation factor A (SII)-like 7 | 492 |
| TCF24 | transcription factor 24 | 460 |
| MAGEA9 | melanoma antigen family A9 | 305 |
| HCN4 | hyperpolarization activated cyclic nucleotide gated potassium channel 4 | 304 |
| NRROS | negative regulator of reactive oxygen species | 242 |
| FAM230B | family with sequence similarity 230, member B (non-protein coding) | 235 |
| PLGLB2 | plasminogen-like B2 | 216 |
| OXGR1 | oxoglutarate (alpha-ketoglutarate) receptor 1 | 214 |
| CRIP3 | cysteine-rich protein 3 | 203 |
| CLEC4F | C-type lectin domain family 4, member F | 200 |
| CDK15 | cyclin-dependent kinase 15 | 198 |
| CRHR1 | corticotropin releasing hormone receptor 1 | 197 |
| CCKBR | cholecystokinin B receptor | 188 |
| WFIKKN2 | WAP, follistatin/kazal, immunoglobulin, kunitz and netrin domain containing 2 | 184 |
| HAAO | 3-hydroxyanthranilate 3,4-dioxygenase | 179 |
| CGB1 | chorionic gonadotropin, beta polypeptide 1 | 176 |
| ALDH8A1 | aldehyde dehydrogenase 8 family, member A1 | 175 |
| PRAP1 | proline-rich acidic protein 1 | 169 |
| HBQ1 | hemoglobin, theta 1 | 166 |
| CACNA1B | calcium channel, voltage-dependent, N type, alpha 1B subunit | 161 |
| OLFML3 | olfactomedin-like 3 | 154 |
| FAM159B | family with sequence similarity 159, member B | 154 |
| NTF4 | neurotrophin 4 | 152 |
| NT5C1A | 5'-nucleotidase, cytosolic IA | 140 |
| TF | transferrin | 132 |

**Supplementary Table 1D: Top 30 down regulated transcripts for ERβ2**

| Gene_ID | NAME | Foldchange |
| --- | --- | --- |
| PVRIG2P | poliovirus receptor related immunoglobulin domain containing 2, pseudogene | -194 |
| PLGLB1 | plasminogen-like B1 | -109 |
| SYT9 | synaptotagmin IX | -73 |
| EN1 | engrailed homeobox 1 | -38 |
| IL18R1 | interleukin 18 receptor 1 | -34 |
| AR | androgen receptor | -33 |
| PCDHB2 | protocadherin beta 2 | -31 |
| C18orf32 | chromosome 18 open reading frame 32 | -18 |
| CRMP1 | collapsin response mediator protein 1 | -18 |
| ACTL8 | actin-like 8 | -12 |
| ZNF883 | zinc finger protein 883 | -12 |
| SAA1 | serum amyloid A1 | -12 |
| ZMYM3 | zinc finger, MYM-type 3 | -8 |
| CASP10 | caspase 10, apoptosis-related cysteine peptidase | -7 |
| SAT1 | spermidine/spermine N1-acetyltransferase 1 | -6 |
| NEFL | neurofilament, light polypeptide | -6 |
| SYT17 | synaptotagmin XVII | -6 |
| HIST3H2A | histone cluster 3, H2a | -6 |
| SPOCK3 | sparc/osteonectin, cwcv and kazal-like domains proteoglycan (testican) 3 | -5 |
| LONRF2 | LON peptidase N-terminal domain and ring finger 2 | -5 |
| PRH1-PRR4 | NA | -5 |
| LINC01234 | long intergenic non-protein coding RNA 1234 | -5 |
| ZNF85 | zinc finger protein 85 | -5 |
| CCNYL2 | cyclin Y-like 2, pseudogene | -5 |
| GADD45B | growth arrest and DNA-damage-inducible, beta | -4 |
| C11orf52 | chromosome 11 open reading frame 52 | -4 |
| SLC16A14 | solute carrier family 16, member 14 | -4 |
| PRRG1 | proline rich Gla (G-carboxyglutamic acid) 1 | -4 |
| HSD17B10 | hydroxysteroid (17-beta) dehydrogenase 10 | -4 |
| CCDC22 | coiled-coil domain containing 22 | -4 |

**Supplementary Table 1E: Top 30 up regulated transcripts for ERβ5**

| Gene_ID | NAME | Foldchange |
| --- | --- | --- |
| NUDT4P2 | nudix (nucleoside diphosphate linked moiety X)-type motif 4 pseudogene 2 | 2559 |
| MAGEB2 | melanoma antigen family B2 | 1736 |
| CHRDL1 | chordin-like 1 | 596 |
| PRIMA1 | proline rich membrane anchor 1 | 589 |
| FLI1 | Fli-1 proto-oncogene, ETS transcription factor | 524 |
| IGSF1 | immunoglobulin superfamily, member 1 | 486 |
| FOXI3 | forkhead box I3 | 411 |
| PRKCB | protein kinase C, beta | 379 |
| TMEM179 | transmembrane protein 179 | 371 |
| PPAPDC1A | phosphatidic acid phosphatase type 2 domain containing 1A | 365 |
| PTCHD4 | patched domain containing 4 | 349 |
| ADAMTS5 | ADAM metallopeptidase with thrombospondin type 1 motif, 5 | 324 |
| SLC6A11 | solute carrier family 6 (neurotransmitter transporter), member 11 | 306 |
| RXRG | retinoid X receptor, gamma | 299 |
| SLC6A17 | solute carrier family 6 (neutral amino acid transporter), member 17 | 295 |
| ARMCX2 | armadillo repeat containing, X-linked 2 | 290 |
| LOC389895 | NA | 264 |
| MRVI1 | murine retrovirus integration site 1 homolog | 259 |
| NPTX2 | neuronal pentraxin II | 257 |
| PLXNA4 | plexin A4 | 256 |
| GPR27 | G protein-coupled receptor 27 | 255 |
| SYT14 | synaptotagmin XIV | 249 |
| BARX1 | BARX homeobox 1 | 247 |
| RGS6 | regulator of G-protein signaling 6 | 243 |
| PDGFD | platelet derived growth factor D | 240 |
| GSC | goosecoid homeobox | 237 |
| GFRA2 | GDNF family receptor alpha 2 | 227 |
| BAALC | brain and acute leukemia, cytoplasmic | 226 |
| TLX3 | T-cell leukemia homeobox 3 | 215 |
| USP51 | ubiquitin specific peptidase 51 | 212 |

**Supplementary Table 1F: Top 30 down regulated transcripts for ERβ5**

| Gene_ID | NAME | Foldchange |
| --- | --- | --- |
| PHF11 | PHD finger protein 11 | -27 |
| GABRB3 | gamma-aminobutyric acid (GABA) A receptor, beta 3 | -9 |
| JMJD7-PLA2G4B | JMJD7-PLA2G4B readthrough | -7 |
| TJP3 | tight junction protein 3 | -5 |
| GS1-259H13.2 | NA | -5 |
| GPER1 | G protein-coupled estrogen receptor 1 | -4 |
| GAS2L1 | growth arrest-specific 2 like 1 | -4 |
| PDE3A | phosphodiesterase 3A, cGMP-inhibited | -4 |
| MRPL45P2 | mitochondrial ribosomal protein L45 pseudogene 2 | -4 |
| ATP8B3 | ATPase, aminophospholipid transporter, class I, type 8B, member 3 | -3 |
| DMGDH | dimethylglycine dehydrogenase | -3 |
| CCDC7 | coiled-coil domain containing 7 | -3 |
| ASB14 | ankyrin repeat and SOCS box containing 14 | -3 |
| RPL35A | ribosomal protein L35a | -3 |
| MFI2 | antigen p97 (melanoma associated) identified by monoclonal antibodies 133.2 and 96.5 | -3 |
| POF1B | premature ovarian failure, 1B | -3 |
| ACY1 | aminoacylase 1 | -3 |
| PLSCR3 | phospholipid scramblase 3 | -3 |
| DNAJC5G | DnaJ (Hsp40) homolog, subfamily C, member 5 gamma | -3 |
| FAM231D | family with sequence similarity 231, member D | -3 |
| HSPA6 | heat shock 70kDa protein 6 (HSP70B') | -3 |
| HYAL2 | hyaluronoglucosaminidase 2 | -3 |
| GTF2I | general transcription factor IIi | -3 |
| GUSBP2 | glucuronidase, beta pseudogene 2 | -3 |
| PHF7 | PHD finger protein 7 | -3 |
| B3GNT9 | UDP-GlcNAc:betaGal beta-1,3-N-acetylglucosaminyltransferase 9 | -3 |
| IFRD2 | interferon-related developmental regulator 2 | -3 |
| LRRCC1 | leucine rich repeat and coiled-coil centrosomal protein 1 | -3 |
| GTF2IRD2 | GTF2I repeat domain containing 2 | -3 |
| KLHL31 | kelch-like family member 31 | -3 |
